# H_2_ Chemical Bond in a High-Pressure Crystalline Environment

**DOI:** 10.1021/acs.jpcc.3c02366

**Published:** 2023-07-31

**Authors:** Miriam Marqués, Miriam Peña-Alvarez, Miguel Martínez-Canales, Graeme J. Ackland

**Affiliations:** Centre for Science at Extreme Conditions and School of Physics and Astronomy, University of Edinburgh, Edinburgh EH9 3FD, U.K.

## Abstract

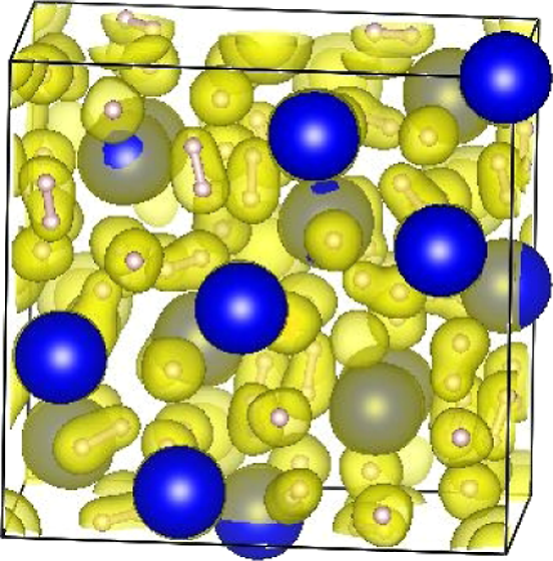

We show that the hydrogen in metal superhydride compounds can adopt two distinct states—atomic and molecular. At low pressures, the maximum number of atomic hydrogens is typically equal to the valency of the cation; additional hydrogens pair to form molecules with electronic states far below the Fermi energy causing low-symmetry structures with large unit cells. At high pressures, molecules become unstable, and all hydrogens become atomic. This study uses density functional theory, adopting BaH_4_ as a reference compound, which is compared with other stoichiometries and other cations. Increased temperature and zero-point motion also favor high-symmetry atomic states, and picosecond-timescale breaking and remaking of the bond permutations via intermediate H_3_^–^ units.

## Introduction

Recent reports of superconductivity approaching room temperature^1−5^ have galvanized attempts to synthesize hydrogen-rich materials. Much effort is being invested to understand their crystal structures and properties.^6−13^ However, high-temperature hydride superconductivity is associated not with a particular crystal structure but with the stabilization of dense atomic hydrogen over molecular dimers.^14−16^ In this paper, we address the fundamental issue of whether hydrogen in metal hydrides is atomic or molecular.^17−19^ Hydrides provide a unique challenge for structural studies, especially under high pressure. From the experimental side, a major challenge is the very weak X-ray scattering by hydrogen atoms. Spectroscopy can only give indirect information about the atomic structure, e.g., a Raman signal related to H_2_ molecules, whose shift can be related to their intra-molecular bond length.

Density functional theory (DFT) is the dominant theoretical method applied for structural determination in these materials. An implicit assumption here is that the lowest-enthalpy static arrangements of the ions will be good candidates for the lowest Gibbs free energy state. In static relaxations, the point symmetry of molecules may constrain the molecular orientation in a given crystal symmetry. However, in practice, due to quantum or classical rotation, H_2_ molecules may behave as spherical rather than linear objects, which in turn allows high-symmetry crystals.^20^

Molecular hydrogen is understood as two hydrogen atoms joined by a covalent bond. In compression of pure hydrogen, the H–H bond lengthens from 0.718 at 6 GPa in phase I to 0.84 Å when entering phase IV at 225 GPa.^21−23^ Although there is no unique definition of a covalent bond in DFT when applied to periodic systems, many measures can be indicative. For example, a well-defined interatomic distance which persists long enough for the bond to oscillate; a high value of the electron localization function (ELF) between atoms; a buildup of charge in the region between the atoms; a narrow Kohn–Sham band of energies, which when projected onto localized basis functions, is localized on the bond. We will utilize all these methods to build a coherent picture of the presence or absence of covalent bonds in a binary hydride solid.

Stable alkaline-earth metals (marked as M hereafter) are known to form binary hydrides (Figure 2). These satisfy the octet rule, and no molecular hydrogen is expected.^24−28^ Tetrahydrides have been experimentally reported for Ca, Sr, and Ba, containing their binary hydride or salt (divalent metal atom, M^2+^, anionic hydrogens, H^–^), and molecular hydrogen, which was interpreted as slightly charged, H_2_^δ−^.^29^ Ca^18,30,31^ and Sr^18,32^ tetrahydrides adopt the *I*4/*mmm* structure, both with *c*/*a* ratios around 1.6, while BaH_4_ forms an *I*4/*mmm* structure with a *c*/*a* of 2.2 as well as an fcc structure.^18^ Raman spectroscopy supports H–H bonds in these tetrahydrides.^18,29,30^ Moreover, the synthesis of *Pm*3̅*n* Ba_8_H_46_ (BaH_5.75_) at 50 GPa,^33^ and *Cmc*2_1_ BaH_12_ at 90 GPa^19^ has also been reported. Neither compound has an identifiable Raman contribution from the stretching H_2_ mode. Therefore, barium hydrides represent an ideal system to study how the change in stoichiometry alters the H_2_ molecule within the solid.^17−19^ Thus, a systematic investigation of the mechanisms involved in hydrogen intra-molecular bond weakening and the influence of the metallic atomic number is here undertaken as the route to understand the concepts of bond-weakening and chemical pre-compression.

We have run an extensive series of DFT calculations. Electronic structure is explored using the ELF and Bader (Quantum Theory of Atoms in Molecules, QTAIM^34^) topologies. We chose *I*4/*mmm*-BaH_4_ as the reference compound because it exhibits both atomic and molecular hydrogen in the same structure. We compare it to other BaH_4_ candidate structures and to other barium hydride compositions such as BaH_2_, Ba_8_H_46_, and BaH_12_. We also contrast it with hypothetical *I*4/*mmm* tetrahydrides of adjacent elements Cs, La, Sr, and Ra.

The paper is organized as follows: The “Methods” section briefly outlines the standard computational approach, before focusing on two key ideas necessary for this work—fictitious mass molecular dynamics and definitive identification of hydrogen molecules. The “Results” section first demonstrates that the concept of a hydrogen molecule in a metal is well defined in theory and calculations. Quantitative results from alkali-earth hydrides are then analyzed; first from a static calculations, followed by an analysis of molecular dynamics trajectories. Further discussion of the various barium hydrides follows, concluding the section with some remarks on the molecular dynamics simulations performed.

## Methods

For static relaxations and ab-initio MD, we carried out density functional theory calculations using the CASTEP code. ELF and Bader analyses were carried out using the CRITIC2 program,^35^ and applied to all single-unit cell static structures. Wannier analysis was carried out using the Wannier90 code,^36^ analyzing DFT calculations performed with Quantum-Espresso^37^ and ultrasoft pseudopotentials.^38^ We also note that the *k*-point convergence of MD calculations is slow.^39^ Full details are given in the Supporting Information.

### Fictitious Mass Molecular Dynamics

It would be prohibitively expensive to run accurate simulations of phase transformations or melting with conventional MD because the Ba atoms are 137 times heavier than hydrogen and move too slowly. Therefore, an efficient way to sample the phase space is to run ab initio molecular dynamics in CASTEP with fictitious masses, *NPT* ensembles, and ringing-mode quenching^40^ (Details Supporting Information 2.2, Table S3).

Our simulations are classical, and our goal in MD is to sample the phase space corresponding to each structure. The partition function for classical interacting particles does not depend on their mass. Therefore, *g*(*r*) and electronic DoS are independent of the choice of mass. We therefore set the metal ion mass similar to hydrogen, which enables much better sampling of the phase space and potentially the discovery of distortions in the metallic sublattice. The drawback is that dynamical properties, such as phonon eigenvectors and diffusion rates are not correct, although mean squared displacement (MSD) can still be used to distinguish solids from liquids.

The MD runs are analyzed using radial distribution functions, lattice parameters, mean squared displacements, and *ab oculo* using VESTA and VMD.^41,42^ After each MD run, snapshots from the trajectory were relaxed. These provided low-symmetry structure candidates and allowed us to validate the bond assignment using ELF or charge density methods. Full details of the molecular dynamics runs and their analysis are given in the Supporting Information Table S2.

### Defining an H_2_ Molecule

The easiest approach to identify a bond is to define a cutoff distance, *R*_bond_, and declare *any* pair of hydrogen atoms closer than this as *“bonded”*. A consistent identification of a molecular bond through an MD run requires *R*_bond_ to be large enough to avoid breaking and reforming the same bond through typical oscillations of the molecule. Based on the observation of a minimum in radial distribution functions, *R*_bond_ = 1 Å is a pragmatic choice. We do not draw conclusions which are highly sensitive to this choice. We further refine our definition by counting the number of atoms with precisely one neighbor within 1 Å, and dividing by 2 to get the number of molecules. In practice, we find that bond-breaking usually occurs via the approach of a third atom and the formation of an intermediate  complex, a process which, by our definition, counts as one molecule throughout. Nevertheless, we record every instance where an H–H distance passes through the 1 Å threshold—this is used for an estimate of bond lifetimes. Partly due to fictitious mass MD, such a lifetime estimate is not quantitatively meaningful (for e.g., calculating Raman linewidths^43−45^) but it aids comparisons between compounds and structures. A detailed discussion of the technical definition of a bond is given in the Supporting Information.

### Identification of an H_2_ Molecule Based on the Electronic Structure

In static relaxation, including from MD snapshots, other measures from the electronic structure can be applied to detect molecules. In *k*-space, a discrete set of electron bands may represent the bond, and this can be tested using a projection of the Kohn–Sham bands onto maximally localized Wannier functions. On the other hand, in real space, and within Bader topology, H_2_ molecules are identified by the presence of a covalent bond between the hydrogen atoms. It is characterized by the high value of the electron density and the negative sign of the laplacian at the bond critical point (first-order saddle point of the electron density connecting the two maxima located on the atoms) (see Supporting Information). The charge of the atoms forming a molecule can be unambiguously calculated by the integration of the electron density within the topological atoms forming the molecule. They are defined by the union of the electron density maxima (located on the atoms) with their attraction basins and delimited by perfectly defined surfaces obeying the zero-flux condition for the electron density.

ELF is a relative measure of the electron localization with respect to the homogenous electron gas. In general, the ELF value approaches 1 in regions of the space where electron pairing occurs (e.g., atomic shells, bonds, and lone pairs). In analogy with QTAIM, a partition of the space based on the ELF can be performed. It consists of non-overlapping basins with well-defined chemical interpretations (cores, bonds, lone pairs). Moreover, the basin populations come from the integration of the electron density within these regions. The signature of a H_2_ molecule within the ELF topology is the existence of a high ELF isosurface enclosing both atoms. Typically, a H_2_ molecule is considered to exist if the minimum ELF value between the two hydrogen atoms is above 0.85. We find these methods to be consistent with one another and with the much faster bond length criterion.

## Results

### Preamble: Hydrogen Molecules in Free Electron Gas

**Figure 1:**
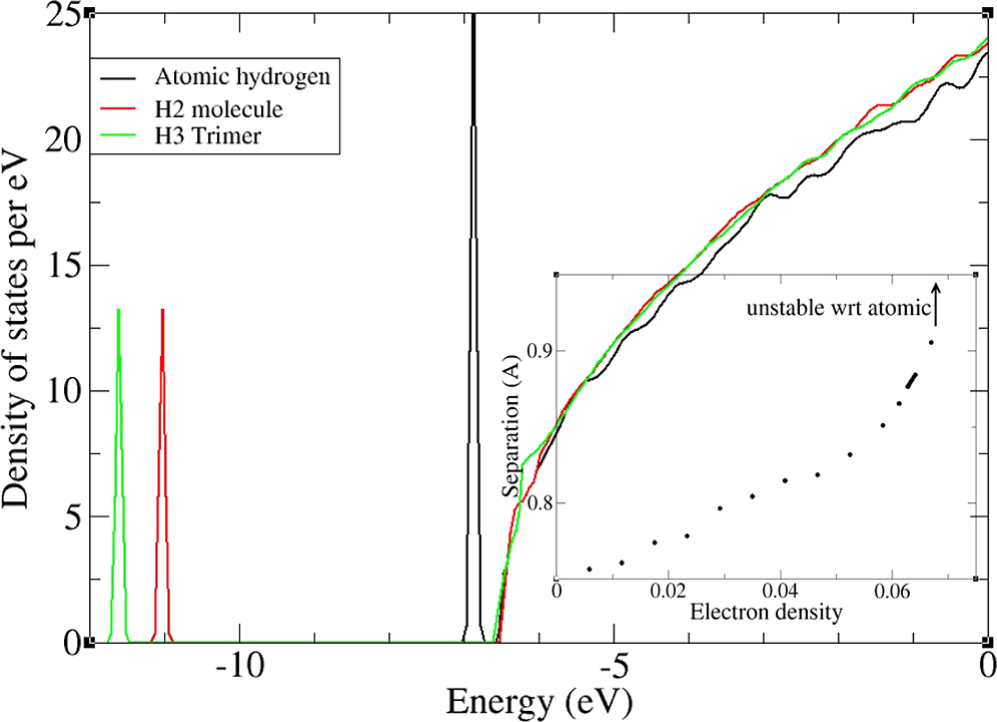
Main figure shows the density of states in jellium for 160 electrons in a 14 Å cube box, plus two or three hydrogen atoms. Both molecular and atomic DoS are shown for the two hydrogen case. The sharp peaks correspond to localized two-electron states and are broadened for clarity. Inset shows the variation of H_2_ bond length with increased electron density (electrons per Å^3^). Wiggles in the free electron section are artifacts from the finite box size.

Breaking the H_2_ bond may occur due to mechanical pressure or electronic effects. To illustrate the electronic effects in isolation, we calculated the hydrogen atom, molecule, and trimer in a homogeneous electron gas,^46^ (Supporting Information 4.3).For low electron density, the band structure comprises a clearly defined H_2_ bonding state and a free electron-like density of states, which lies much higher in energy (Figure 1). At electronic densities above 0.06 *e*/Å^3^ (*r*_s_ ≈ 3.0 *a*_0_), the bond breaks spontaneously. This is evidenced by a discontinuous jump in the H–H separation, the vanishing of the flat Kohn–Sham band defining the molecule, and the appearance of atomic-hydrogen (H^–^) states at the bottom of the free-electron band. This suggests an upper limit on the electron density where molecular hydrogen can be found. Interestingly, the maximum bond length before the transition is less than 0.9 Å.

The picture which emerges from this preliminary study is that hydrogen in metals can exist as H_2_ molecules or as H^–^. These are in competition, and they are distinct. Considering the electron’s energy relative to the Fermi surface, H_2_ accommodates two electrons bound by about −11 eV each. By contrast, the atomic form 2H^–^ accommodates four electrons bound just below the bottom of the free electron band. As the electron density increases and the band broadens, the atomic state becomes favored over the molecular one. This essential competition between atomic and molecular states occurs in all materials considered.

We also calculated the H_3_^–^ linear trimer: this can be understood as a particle in a box or via molecular orbitals (MO). The bonding state in the particle-in-a-box picture has the electron delocalized across three atoms. In MO theory, it would be written as (ϕ_s1_ + ϕ_s2_ + ϕ_s3_) with ϕ_sx_ the s-orbital on the *x*th atom. The next state is the antisymmetric first excited state of the particle-in-a-box with a node on the central atom, or in MO theory, the ungerade non-bonding state (ϕ_s1_ – ϕ_s3_). This is typically occupied and is hybridized with the free electron band. The third state has a maximum on the central atom and in MO can be viewed as a gerade antibonding state: (ϕ_s1_ – 2ϕ_s2_ + ϕ_s3_). It is typically unoccupied.

There is no explicit link to pressure in this calculation, but note that one way to increase electron density is by reducing the volume. The implication is that alloying elements with high valency and small volumes (e.g., Y, La, Ac, Lu) will favor atomic hydrogen over the molecular form.

We can conclude from this that H^–^, H_2_, and  are well-defined objects within a free electron gas. The energy of the bonding state in H_2_ and  is broadly independent of the electron density, whereas the H^–^ has an energy at the bottom of the free energy band. Consequently, the molecular forms become unstable at a high enough electron density when the width of the free electron band is approximately *half* the bond energy—this is simply because two H^–^ ions can accommodate twice as many electrons as one H_2_ molecular bond.

### Molecular and Atomic H in Static Calculations

**Figure 2:**
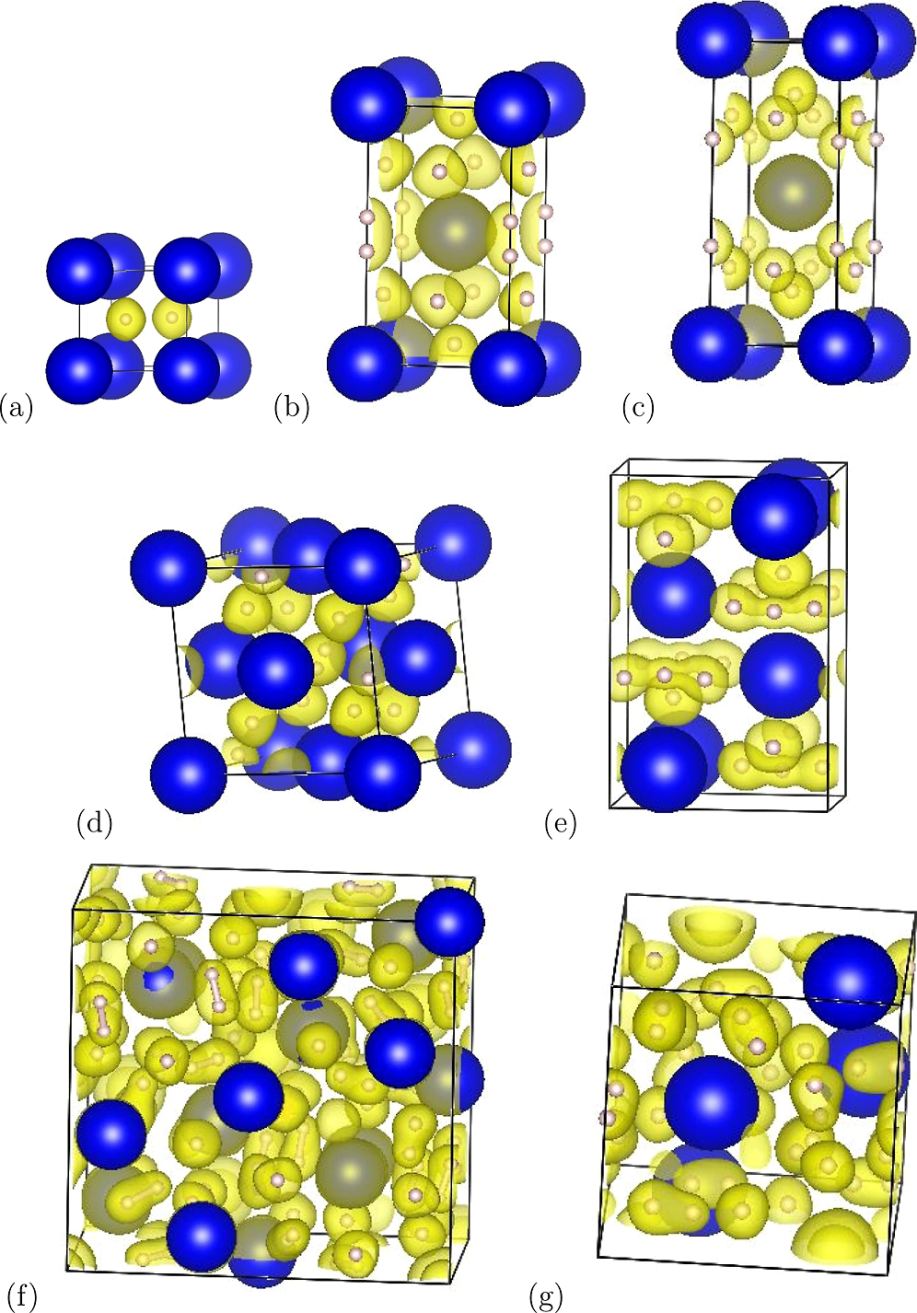
Crystal structures with Ba and H atoms represented as blue and pink spheres, respectively. ELF isosurfaces (ELF = 0.85) shown in yellow are associated with anionic hydrogens and molecules. (a) *P*6/*mmm*-BaH_2_, (b) *I*4/*mmm*-BaH_4_ (low *c*/*a*), (c) *I*4/*mmm*-BaH_4_ (high c/a), (d) *R*3̅*m*-BaH_4_, (e) *Cmcm*-H3-BaH_4_, (f) *Pc*-BaH_5.75_, and (g) *P*2_1_-BaH_12_ at 50 GPa. Other structures are shown in Figures S8–S12.

We begin our analysis of alkali-earth tetrahydrides by focusing on various competitive structures found in our previous extensive searches and compatible with our experimental results.^18^ These include crystal structures of symmetry *I*4/*mmm* (two *c*/*a* ratios), *Cmcm*, *C*2/*c*_*,*_ and *R*3̅*m*. Their details will be discussed later in this section, and the most important ones are shown in Figure 2. For each of these structures, we performed a full geometry optimization at various pressures. Here, we focus on 50 GPa. It should be emphasized that, while our structure searches identify lower-enthalpy BaH_4_ structures in this pressure range, their X-ray diffraction pattern is incompatible with the experiment. Table 1 shows the main structures, together with their metallic character and an analysis of the atomic/molecular characteristics of hydrogen.

**Table 1:** Calculated PBE Properties of *I*4/*mmm*-XH_4_ Structures at 50 GPa and Five Other Structures^18^^a^

system	*c*/*a*	nearest H–H, Å	volume	o,t ELF basin population	metal
BaH_4_*Cmcm* H_2_	1.716	0.806	68.00		no
BaH_4_*Cmcm* H_3_	1.469	0.944 (H_3_^–^)	68.12		no
BaH_4_*C*2/*c*	1.514	0.829	70.87		no
BaH_4_*C*2/*c* I	1.467	0.942 (H_3_^–^)	68.17		no
BaH_4_*C*2/*c* II	2.000	0.822	68.756		no
BaH_4_	n/a	0.831	72.44		no
BaH_4_ (low)	1.763	0.803	72.28	1.047 1.731	no
BaH_4_ (high)	2.323	1.495	65.96	1.367 1.337	yes
SrH_4_ (low)	1.712	0.798	61.42	1.050 1.772	no
SrH_4_ (high)	2.083	1.629	57.14	1.362 1.416	yes
RaH_4_ (low)	1.721	0.796	78.58	1.052 1.773	no
RaH_4_ (high)	2.338	1.655	72.22	1.390 1.362	yes
CsH_4_ (low)	1.642	0.768	69.38	1.029 1.451	yes
CsH_4_ (high)	2.821	1.489	67.98	1.258 1.098	yes
LaH_4_	1.863	1.502	64.56	1.450 1.604	yes

a Here, “high” and “low” refer to the initial *c*/*a* ratio before relaxation being about 2.2 and 1.6, respectively. Atomic ELF populations are centered on the octahedral site (forming two independent atoms or a molecule) and located on the tetrahedral site, respectively. LaH_4_ exhibits only one minimum.

### Ubiquitous *I*4/*mmm* Structure

X-ray diffraction measurements report that the cations in a number of tetrahydrides adopt the same structure, with the cation occupying the 2*a* Wyckoff orbit in the *I*4/*mmm* space group.^18,29^ For Sr, Ba, Ra, and Cs *I*4/*mmm* tetrahydrides, we find two energy minima in *I*4/*mmm* as a function of the *c*/*a* ratio (Table 1). Despite having the same symmetry, there is a clear distinction between the two, with very different *c*/*a* ratios, H–H separations, and metallic character. The high *c*/*a* version (*c*/*a* ∼ 2–2.8) is metallic and has atomic H^–^. The low *c*/*a* version (*c*/*a* ∼1.7) has molecular H_2_ and is semiconducting in alkaline earths but metallic in Cs and La. Typically, the atomic version has a higher density and becomes stable at higher pressure.

**Figure 3:**
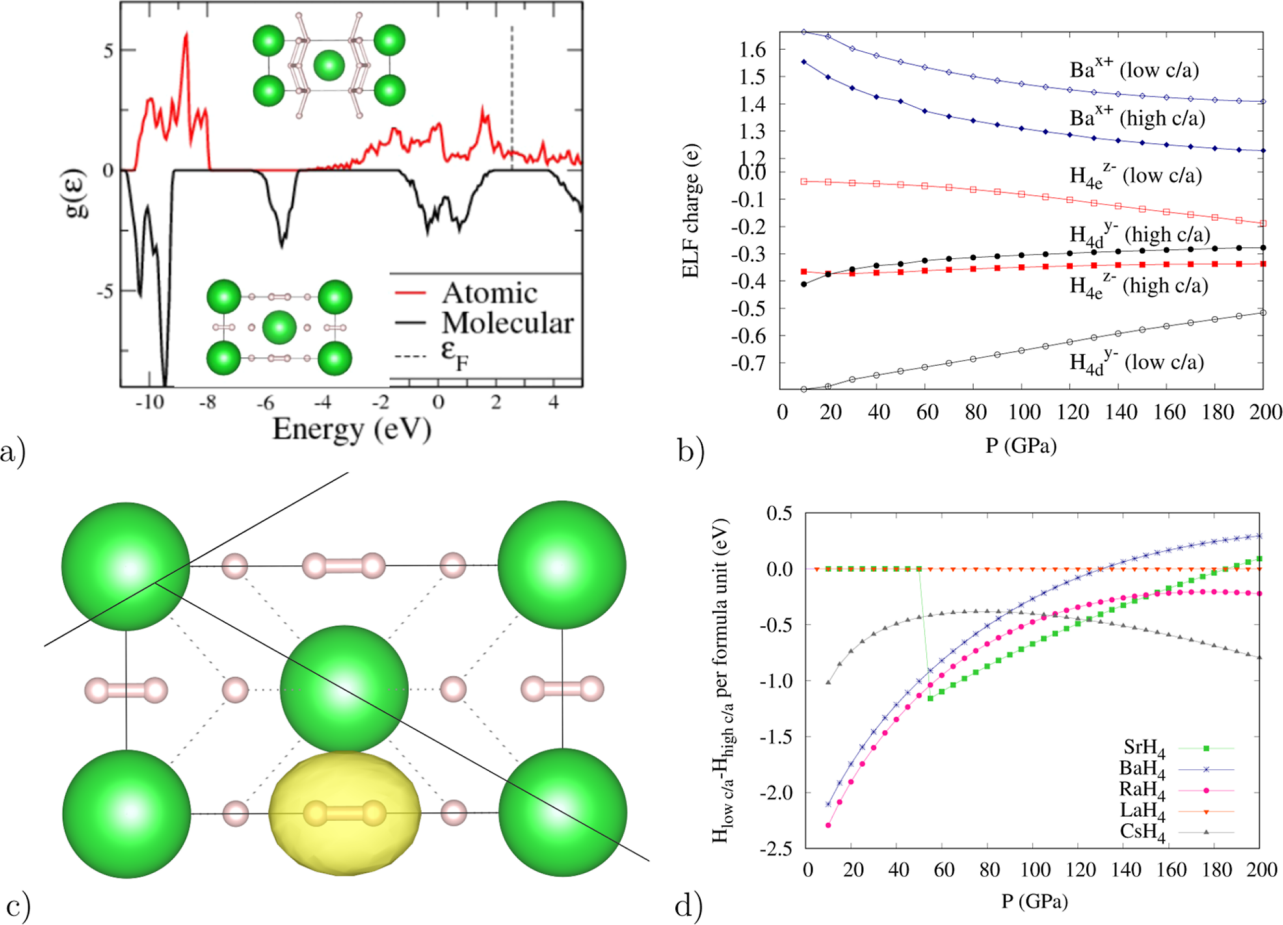
Results of DFT calculations for the isostructural forms of *I*4/*mmm*-BaH_4_. Atomic *I*4/*mmm*-BaH_4_ has a high c/a ≈ 2.40; closest hydrogen pairs are ≈1.5 Å apart in the *xz* plane. Molecular *I*4/*mmm*-BaH_4_ has a low c/a ≈ 1.8 at 100 GPa: bonds are along *z*-direction <0.85 Å (a) Density of electronic states at 10 GPa (b) charges based on ELF topology. (c) Maximally Localized Wannier Function whose projection corresponds to the covalent band (d) enthalpy differences between high *c/a* and low *c/a* for various *I*4/*mmm* compounds, n.b. LaH_4_ has only one minimum at low pressure.

Figure 3 and Tables 1 and S1 show the two *I*4/*mmm* structures, ELF populations, and Bader charges. All hydrogens in the high c/a phases have broadly similar charges, whereas in the low c/a phases, the molecular hydrogens are essentially neutral (the integration of the electron density within the H_2_ units according to both Bader and ELF topologies gives a value close to 2 electrons), whereas atomic hydrogens have a sizeable negative charge. The evolution of the ELF charges with pressure is shown in Figures 3b and S4. In low c/a structures, in all compounds, a narrow band of states around 6 eV below the Fermi energy is clearly associated with the H_2_ molecules (Figure 3). Projecting that band onto maximally localized Wannier functions (Figure 3c) shows this band to be primarily associated with a H–H σ bond.

The full band structures for all *I*4/*mmm* compounds at 50 GPa are shown in Figure S7, where the “molecular” bands are conspicuously absent for the Lanthanum compound: the trivalent ion donates sufficient electrons to disrupt the molecule formation.

Finally, Figure 3d shows the enthalpy cross-over from the low-*c*/*a* to the high *c*/*a* with pressure (zero in LaH_4_ below 50 GPa, where only a single minimum exists).

### Other BaH_4_ Structures

**Figure 4:**
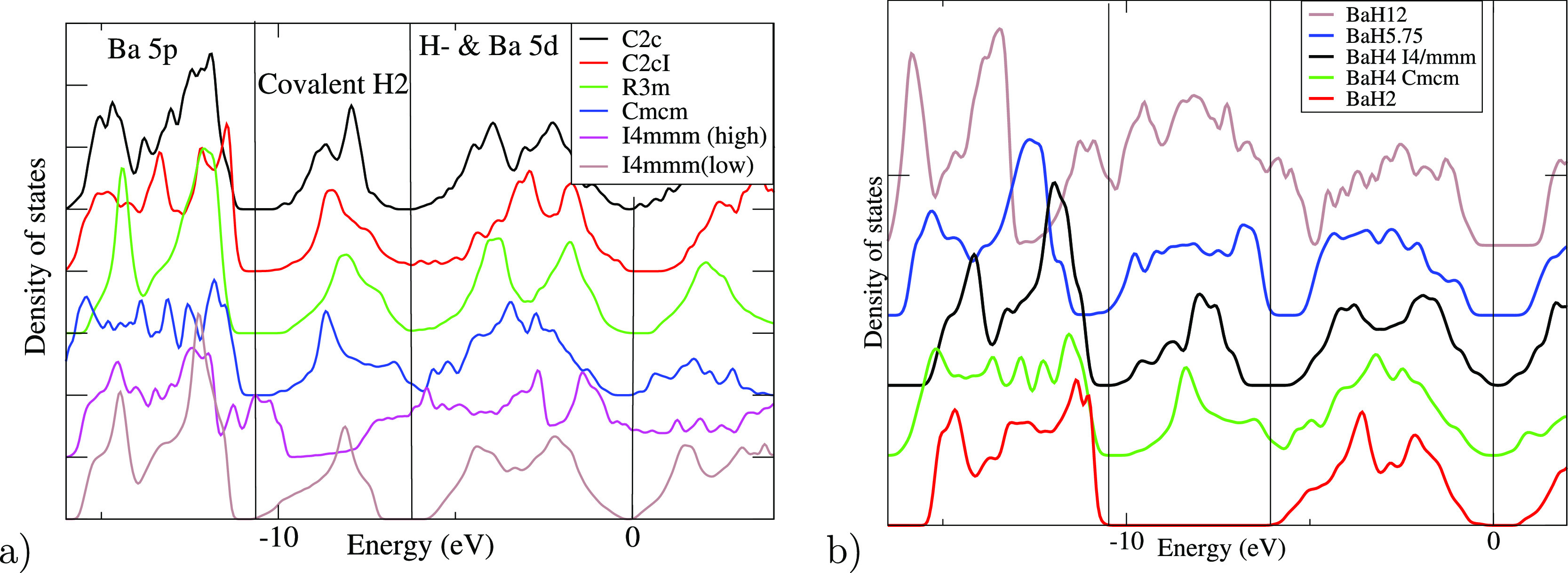
Density of electron states from statically relaxed Barium hydrides at 50 GPa normalized to  calculated using PBE. (a) BaH_4_ candidates, with four distinct bands associated with their largest projection onto localized states Ba 5*p*, H_2_, Ba 5*d* + H, and Ba 6*s* + 5*d*. (b) Other stoichiometries. *Cmcm* refers to *Cmcm*-H_2_. Note that the BaH_2_*P*6_3_/*mmc* (red line) has no H_2_ molecules and no density of states in that region.

In a previous work, we reported structure search calculations^18,47^ which identified other possible stable structures for BaH_4_. In BaH_4_ the static *I*4/*mmm* calculation favors the low *c*/*a* ≈1.76, but other BaH_4_ structures are calculated to be more stable than any *I*4/*mmm*^18^ (see Supporting Information). Some of these are similar to *I*4/*mmm*, e.g., *R*3̅*m* is simply a rotation of the hydrogen molecules out of *I*4/*mmm* symmetry, leaving Ba in very similar positions. However, two structures with *Cmcm* symmetry have especially low enthalpy. These differ in enthalpy by only a few meV at 50 GPa, but are chemically very different (*Cmcm*-H_2_ lattice angle γ = 141° with H_2_ molecules and *Cmcm*-H_3_ γ = 114° with H_3_^–^ units, see Figure 2). The *Cmcm*-H_3_ version is more stable with the PBE and BLYP functionals, whereas LDA favors *Cmcm*-H_2_. Both *Cmcm* structures contribute two e^–^/f.u. to the “H_2_” band (Figure 4). The *Cmcm*-H_3_ contains linear H_3_^–^ trimeric ions. The topological charges are shown in Table S2. The anionic character of H_3_^–^ is clear. This unit can be assumed to have four electrons, two in symmetric bonding states with energies similar to the H_2_ band, and two in antisymmetric “nonbonding” states with energies similar to H^–^

In any case, these structures are very different from either of the experimentally reported structure for BaH_4_.^18^

### Other Barium Hydride Compositions

While BaH_4_ has equal numbers of atoms and molecules, other compositions of barium hydride may have different ratios.

BaH_2_ is an ionic compound based on H^–^ ions in interstitial positions in hcp Ba. It transforms from distorted orthorhombic *Pnma* to symmetric hexagonal *P*6_3_/*mmc* at high pressure and temperature.

BaH_5.75_ adopts the Weaire–Phelan structure, a so-called topologically close packed phase in which all interstitial sites are tetrahedral. The fractional stoichiometry arises from locating one hydrogen ion in each tetrahedral interstitial site.

BaH_12_ is a superhydride, with Ba located in cages of hydrogen atoms.

### Band Structure in Barium Hydrides

Figure 4 shows a remarkable similarity in the electronic density of states between the BaH_4_ candidates and other barium hydrides of various compositions. The extra electrons from hydrogen-rich stoichiometry are all located in the H_2_ band. Projections of the wavefunctions onto atomic orbitals enable us to define distinct characteristics for well-defined groups of bands, independent of crystal structure. For BaH_4_, distinct bands can be associated with Ba 5p, molecular hydrogen, and atomic hydrogen. All these solids are small-gap semiconductors. This suggests that even at 50 GPa, all these compounds can be regarded as . This does not hold for *I*4/*mmm* with high *c*/*a*, which has no molecules and no associated peak in Figure 4 and is highly unstable in MD.

### ELF and Charge Density

High ELF values are found at the bond points connecting all pairs of hydrogen atoms separated by < 1 Å (Figures 2 and S15). If the bands localized in energy space at around −6 to 10 eV below the Fermi energy are projected onto maximally localized Wannier functions, they are observed to also be localized in real space.

### Comparative Molecular Dynamics of Barium Hydrides

We analyzed the dynamics of the aforementioned systems via MD. As mentioned before, the large mass difference makes it prohibitively expensive to sample phase space using physically correct masses, so instead we performed fictitious mass MD, as detailed in the Methods section and Section S2. The MD runs are analyzed using radial distribution functions (RDF), lattice parameters, mean squared displacements (MSD), and visually inspecting the trajectories. After each MD run, snapshots from the trajectory were relaxed. These provided low-symmetry structure candidates, and allowed us to validate the bond assignment using ELF or charge density methods. Full details from the molecular dynamics runs and their analysis are given in Table S2. Here, we describe features common to all barium hydrides.

The MSD (Figure S17) shows that the hydrogens move much further than barium but quickly flatten off. This shows all materials considered are crystalline up to 600 K, not superionic or liquid. The one exception was BaH_5.75_ at 1000 K, which went through a melting transformation after 1.2 ps (fictitious time).

**Figure 5:**
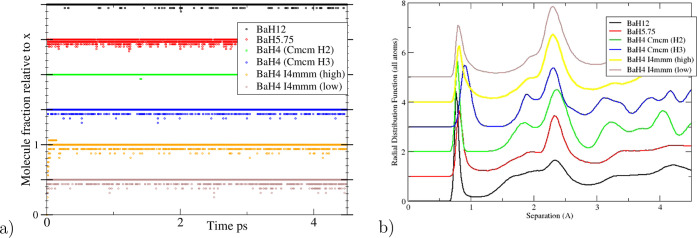
Analysis of MD at 50 GPa/300 K. *I*4*mmm*/fcc indicates simulations in *I*4/*mmm* for which the c/a ratio is close to  (left) Fraction of bonds relative to *x* in as a function of time for various structures. Each line is displaced by 0.5 for clarity, showing that the number of bonds is remarkably close to the appropriate *x* and that deviations are invariably below *x*, during bond-breaking events. (right) Radial distribution functions, normalized to the number of atoms pfu. The large peak at 2.4 Å corresponds to BaH pairs.

Typical RDFs are shown in Figure 5. They all have a well-defined peak between 0.7 and 1.0 Å. We thus selected 1.0 Å as our bond cutoff. Integrating the first peak or counting the number of bonded pairs gives a number of bonds in all cases consistent with , where *x* is the number of bonds required to make up the stoichiometry. This observation holds for BaH_2_, all BaH_4_ structures, BaH_5.75_ and BaH_12_. All peaks in the H–H RDF are in much the same place, regardless of composition (Figures 5 and S16). The shortest Ba–H distance represents atomic size, and the first H–H is as expected for a molecule, but the second H–H peak being similar is surprising. Under pressure, the molecular peak moves to a larger bond length, and subsequent peaks move to a shorter separation as density increases.

We estimate relative molecular lifetimes between structures by counting how many times an H–H distance is exactly 1 Å (See Table S2). In all MD structures, molecules have lifetimes of hundreds of fs, an order of magnitude larger than their vibrational periods. In BaH_4_, the H_2_ molecular version of *Cmcm* has the longest lifetime, while *I*4/*mmm* (high) has the shortest. The bond breaking rate increases with temperature and pressure. Bond breaking is normally mediated by a third hydrogen atom, which forms an H_3_^–^ complex, which then breaks back into a molecule and an isolated atom, which may originally have been in the molecule. These  units are usually transient, but are also observed with two equal H–H distances in MD and static relaxation, e.g., *Cmcm*-H_3_, implying that they represent a stable structural motif. The number of singly bonded atoms remains remarkably stable at 2*x* in all  compounds. It is robust to the choice of bond cut-off, whereas the number of unbonded and double-bonded hydrogens is very system- and time-dependent. Fourfold coordinated hydrogen is never observed.

#### BaH_4_

Turning to specifics, for *I*4/*mmm*, the two distinct *c*/*a* ratios behave very differently. In the low *c*/*a,* the molecules persist at 1 per formula unit (pfu), with an average bond length of 0.81 Å. They rotate continuously, and the *c*/*a* drops well below the static value. To within sampling and finite size error, , which means that in X-ray diffraction from the Ba atoms will be indistinguishable from the reported fcc structure. At 50 GPa and 1000 and 600 K, the unit cell remains orthorhombic, but at 300 K, there is a fluctuation between the low-*I*4/*mmm c*/*a* ∼ 1.75 and the even lower fcc-like *c*/*a* ∼ 1.41. In the high *c*/*a* ratio version, molecularization occurs between atoms in the same *xy* plane, not those sharing the octahedral interstice. At 50 GPa and 300 K, there is one molecule of pfu. At 200 GPa, where static calculations suggest that high *c*/*a* is more stable than low *c*/*a*; our algorithm still detects 0.84 molecules/f.u., but this varies during the run and is sensitive to details of the calculation because these molecules are short-lived and long (0.95 Å on average).

The two *Cmcm* structures are intermediate in density between atomic and molecular *I*4/*mmm*. In molecular dynamics, their hydrogen atoms show no rotation. This offers an immediate explanation for why the *I*4/*mmm*/*fcc* structures are more stable at room temperature: there is a significant rotational contribution to the entropy.

#### BaH_5.75_

BaH_5.75_ has been synthesized experimentally, and the Ba positions were identified via X-ray diffraction to correspond to the Weaire-Phelan (A15 or β-W) structure. This is a topologically close-packed structure  in which all interstitial sites are tetrahedra. Theory suggests that the unusual 1:5.75 stoichiometry comes from placing one hydrogen in each tetrahedron. If the hydrogens are placed in the tetrahedra and relaxed, preserving *Pm*3̅*n* symmetry, the octet rule cannot be satisfied, and precisely one H_2_ molecule per Ba forms. To satisfy the octet rule (1.875 molecules per Ba), much larger supercells are needed, with at least 8 symmetry-inequivalent Ba atoms.

The most stable structure we observe has *Pc* symmetry with 30H_2_ units (identified from both RDF and ELF, Figure 2): . which has 3.75 electron bands per Ba in the “H_2_” region of the bandstructure (Figure 4). The 92 tetrahedra in the Ba_16_H_92_ cell contain 182 triangular faces across which the molecule could form. This gives a large number of possible choices and pairings of 60 out of 92 hydrogen atoms, and associated configurational entropy. Although *Pc* is the most competitive BaH_5.75_ structure we found with 16 Ba atoms, and even though its phonon spectrum indicates stability against small distortions, even larger supercells should not be ruled out. Throughout the MD, bonds form between atoms in adjacent tetrahedra: as a consequence, the H_2_ molecules do not rotate, but the structure is dynamic because bond breaking takes place via  units.

#### BaH_12_

We performed similar MD calculations on pseudocubic BaH_12_,^19^ at pressures up to 150 GPa. We find that any structure consistent with cubic symmetry is, in DFT, highly unstable compared to H_2_ bond formation.

The previous work^19^ identified numerous candidate phases with large supercells and low symmetry. By quenching molecular dynamics, we found a similar plethora of near-stable structures. In both studies, the metastable structures with broken symmetry involve at least four formula units (e.g., Ba_4_H_48_, *P*2_1_ symmetry, or several variants with *P*1).

The common theme of these structures is molecularization, confirmed by ELF analysis (Figure 2): the molecular distances range from 0.77 to 0.9 Å, with a clear gap to the next H–H distance (1.02 Å) and the number of molecules follows the octet rule: . There are also  units appearing in the MD, and in some cases, these persist during relaxation.  is consistent with the octet rule if regarded as closely separated H_2_ + *H*^–^.

Our molecular dynamics approach shows the continuous making and breaking of bonds via  units. As such, the true structure is likely to be a dynamic superposition of these phases. It is difficult and unnecessary to try to identify the exact ground state. Our work is therefore fully consistent with the previous static calculations, adding a chemical logic to the structures found there.

#### BaH_2_

By contrast, BaH_2_ is an unremarkable ionic insulator, with no close H–H distances and no electrons in the energy region associated with H_2_ molecules (Figs.2,4). The ambient cotunnite structure transforms to the Ni_2_In *P*6_3_/*mmc* structure at high temperatures and above 1.6 GPa. At around 50 GPa, it further transforms to a metallic *P*6/*mmm* structure.^48−52^ Molecular dynamics shows no evidence of any molecule formation.

#### Empty Lattices

We also studied the electron density and ELF for calculating “empty” *I*4/*mmm* structures comprising just Ba without any hydrogen, and *I*4/*mmm* BaH_2_ without the hydrogen molecules. We found that for the low *c*/*a* ratio where interstitial hydrogen adopts a molecule form in the octahedral site, there is a single ELF maximum centered on the octahedral site and elongated along the direction of the hydrogens forming the molecule. At low pressures, there is no difference in the ELF value for the associated ELF basin, but at higher pressures, the ELF value at the ends of the basin is slightly higher than that at the octahedral site, signaling the stabilization of the atomic phase at high pressure. Indeed, for the high *c*/*a* phase stable at high pressures and where hydrogen forms two atoms, the original ELF basin is broken in two well-differentiated ELF maxima with a low ELF value in between. Interestingly, these ELF maxima are not non-nuclear maxima of the electron density. Therefore, the ability of high-pressure metals to host molecular hydrogen and their corresponding positions should be understood by resorting to the ELF topology.

Calculations of substoichiometric *I*4/*mmm* showed that the most stable defect is to remove the hydrogen molecule altogether - replacing H_2_ with H in the octahedral site is energetically unfavorable.

## Discussion and Conclusions

We have shown that at intermediate pressures, superhydrides tend to form molecule-containing phases. These types of structures are qualitatively different from the atomic structures, which are associated with superconductivity. Typically, the pairing of the hydrogen atoms and the molecular orientation leads to much lower symmetry than the atomic phases.

This general picture explains why hydride superconductivity has, to date, only been observed at high pressure. The Cooper pairs can form only if the hydrogens are not already paired into molecules since this removes the electrons from deep-lying states.

The atomic superhydride phases, by which we mean more hydrogens per cation than the valency of that cation, are typically favored by high electron density, which means high valencies and pressure.

Localized H_2_ molecular units are well defined in five different metrics: bond length, bandstructure, Wannier wavefunction projection, and in both Bader and ELF topologies. The number of H_2_ molecules is determined by the octet rule BaH_2_(H_2_)_*x*_: with *x* = 0, 1, 1.875, and 5. This value for *x* persists in molecular dynamics of all these materials, even after melting. In related compounds, the octet rule holds, and we observed CaH_2_(H_2_)_*x*_, SrH_2_(H_2_)_*x*_*,* and RaH_2_(H_2_)_*x*_*,* but CsH(H_2_)_*x*_ and LaH_3_(H_2_)_*x*_ are formed in molecular dynamics.

Calculated hydrogen positions for stable structures of BaH_2_(H_2_)_*x*_ are inconsistent with the reported room temperature experimental symmetry. In the case of BaH_2_(H_2_), this can be explained by the MD calculations: molecular rotation in the *I*4/*mmm* structure leads to a *c*/*a* ratio of , for which the Ba positions are at the fcc sites as observed by diffraction. Rotation means that there is no symmetry-breaking due to molecule orientation. The rotation also means *I*4/*mmm* has high entropy, which stabilizes it against the lower-enthalpy *Cmcm* phases, which are predicted to be stable at low temperatures. For the topologically close-packed BaH_5.75_ compound, molecular rotation cannot occur because each hydrogen atom is trapped in its own tetrahedral cage. The bonds are continuously being made and broken - on a picosecond timescale across the face of the tetrahedra, while the Ba atoms remain in positions observed in the experiment. Remarkably, at any given time, the number of such bonds meets the  octet criterion. For BaH_12_, the decomposition into five molecules per unit cell is inconsistent with the available Wyckoff sites in cubic symmetry. Once again, molecular dynamics shows that continuous bond breaking and making occurs, such that a hydrogen is sometimes bonded to each of its neighbors, and on average, the high symmetry structure is recovered.

The instability of high-symmetry structures in forming electron pairs as bonds is likely to compete with the superconductivity instability toward Cooper pair formation. Since our calculations are carried out using Kohn–Sham wavefunctions, Cooper-pair correlations cannot be observed (see Supporting Information 7).

We find that the favored hydrogen positions can be predicted from the ELF maxima of the “empty lattice”. In this sense, hydrogen can be regarded via an “embedded atom” picture rather than forming metal-hydrogen chemical bonds—exactly as proposed some 40 years ago and developed into the embedded-atom method which pervades materials science.^53^ This idea based on the anions in metallic matrices model^54^ was also advanced more recently by Sun and Miao,^55^ who also observed that metal ions stabilize H against H_2_. However, we find no evidence to support their notion that the hydrogen network can usefully be regarded as an aromatic network—in our picture, the H- (or H3-) ions are the relevant building blocks, and our localized Wannier function analysis shows this. In the atomic phases, the “hydrogen-derived” electronic states are simply subsumed into the conduction band. We find that the typical H–H distance in the atomic phases is far too large for direct H–H aromatic bonds to be credible, and we have been unable to find any signatures of this in the electronic structure.

Belli et al.^56,57^ have considered how the network in the atomic phase can be defined using ELF and how this correlates to superconducting temperature. To a first approximation, they show that high-Tc superconductors occur when the free-electron value for ELF = 0.5 of above spans the crystal. Our work extends this insofar as the molecularization we report here entails a breakdown of that network and, consequently, of superconductivity.

We show that there is a lengthening and weakening of the H_2_ bond purely with increased electron density. Since density increases with pressure, this is one pressure-induced mechanism which can be isolated as a contributor. We also found that the maximum bond length, which can be sustained by H_2_ in jellium, is less than that sometimes observed in compounds. Additional effects might be resonance with other hydrogen atoms; typical bond lengths in H_3_^–^ are larger than in H_2_. We showed that molecular hydrogen tends to form in the largest available interstices, so the compression will destabilize the bond. Stable structures containing H_3_^–^ units typically have metal cation arrangements which accommodate these larger objects.

This work reveals the persistence of the H_2_ bond in classical molecular dynamics of high pressure compounds. Molecular rotation and dynamic bond breaking allow this to occur without XRD-detectable long-range symmetry breaking. Furthermore, the requirement from the octet rule to form, e.g., BaH_2_(H_2_)_*x*_, and the incompatibility of *x* with simple Wyckoff sites lead to frustration in the bonding network and lowest-energy structures having large unit cells and very low symmetry. However, at room temperature, H_2_ molecules are being broken on a picosecond timescale through the formation of H_3_^–^ units, and the ensemble-averaged structures may restore the high symmetry of the cation sublattice.

Overall, we show there is a clear distinction between atomic and molecular forms of hydrogen in superhydrides. Under pressure, there is competition between molecularization and atomization, with pressure favoring atomization. Even when the molecular-type phases are stable, symmetrization occurs if all permutations of the H_2_ molecules are formed over short periods of time. The existence of an ensemble of possible bonding states in these classical simulations gives a strong hint that if the nuclei are treated quantum mechanically, the atomic phase can be further stabilized. Also, we found that there is a critical electron density above which H_2_ molecules cannot exist. This competition explains the fact that known hydride superconductors exist only at high compressions, where the atomic form becomes stable against molecularization.

## Supporting Information Available

Further details of the simulations and analysis is available in the Supporting Information. Full trajectories and example low-symmetry relaxed structures comprise many GB of data and are available from the authors on request (PDF)
