# Supplementary material for: H2 Chemical Bond in a High-Pressure Crystalline Environment
Source: J Phys Chem C Nanomater Interfaces. 2023 Jul 31;127(31):15523–32. doi: 10.1021/acs.jpcc.3c02366 (PMC10424234; doi:10.1021/acs.jpcc.3c02366)
Supplement: Supplementary file 1 — jp3c02366_si_001.pdf [file jp3c02366_si_001.pdf]

Supporting Information for: The H_2 chemical bond in a high-pressure crystalline environment

Miriam Marqués, Miriam Peña-Alvarez, Miguel Martínez-Canales, and Graeme J. Ackland**

Centre for Science at Extreme Conditions and School of Physics and Astronomy, University of Edinburgh, Edinburgh EH9 3FD, United Kingdom

E-mail: gjackland@ed.ac.uk

List of Figures

S1 (right) Projection of Kohn-Sham band onto Maximally Localised Wannier function for BaH_4 $I4/mmm$ structure showing localisation on a single H_2 unit. (right) Bandstructure of BaH_4 with colours showing projection onto H_2 covalent bond orbitals. S11

S2 (right) Projection of Kohn-Sham band onto Maximally Localised Wannier function for the *Cmcm*-H3 structure. (a) Band structure: bands are enumerated from lowest energy upward, including the Ba 6s (b) Projection of MLWF onto Bloch states. (c) Projection onto Ba 5p MLWF states. (d) Projection onto atomic H 1s MLWF states (e) Projection onto MLWF states for H_3^- (f) Isosurface of an H_3 MLWF S12

S3 **The maximum electron density into which an H_2 molecule can be embedded is 0.07 Å $^{-3}$.** a) Plot of bondlength vs DFT Calculation using PBE hydrogen embedded in a jellium background at the shown electron density. Calculations were done with one hydrogen molecule in a 343 Å ^{3} box with a 16^3 k–point grid. Similar calculations using BLYP give the bond breaking at the same density b) Bandstructure calculations (density of states) for the system described above with a total of 23 or 24 electrons in the system. The covalent bond in the 23 electron system lies some 10 eV below the Fermi energy, or 4.5 eV below the bottom of the free electron band. After bond-breaking, the atomic states lie at the bottom of the free electron band. Other oscillations in the free-electron band structure come from the cubic symmetry of the supercell and are not relevant. S15

S4 Evolution of c/a with pressure for the $I4/mmm$ structures. Solid and open symbols correspond to the low and high c/a structures. The $I4/mmm$ structures with the lowest enthalpies at each pressure are indicated by solid lines. S17

S5 Evolution of a lattice parameter with pressure for the $I4/mmm$ structures. Solid and open symbols correspond to the low and high c/a structures. The $I4/mmm$ structures with the lowest enthalpies at each pressure are indicated by solid lines. . S18

S6 a) Charges as a function of pressure in $I4/mmm$ compounds based on the ELF topology for the metal and hydrogen atoms located on 4*e* and 4*d* sites S19

S7 Band structures at 50GPa in SrH_4, BaH_4, LaH_4, CsH_4 and RaH_4. The zero of energy has been offset to place the peak of the H_2 "band" together, as indicated by the arrow. The calculated peaks contain 2 (not shown), 6 (double peak, widely separated e.g. in Cs), 2 and 4 (double peak) electrons per formula unit respectively. In BaH_4 these can be interpreted chemically as Ba 5s, Ba 5p, molecular $H_2\sigma$, and atomic H 1s. The Fermi energy lies in the gap at +6 eV and the conduction band has 5d and 6s character. S21

S8 Structures from $I4/mmm$ MD simulations. (left: MD snapshot 50GPa/300K; centre: snapshot after relaxation with H_3^- units; right: 200GPa snapshot after relaxation with H_2 units) S23

S9 **At room temperature both high and low c/a structures of I4/mmm BaH_4 contain molecules** Snapshots from PBE MD simulation of (a) high $<a>= 5.38, <b>= 5.40, <c>= 12.29$ and (b) low MD simulations at 200GPa and 300K $<a>= 5.807, <b>= 5.50, <c>= 11.2$. Analysis of the number of dihydrogen pairs at less than 1Å shows a distinct difference. The low c/a version averages 0.99 molecules per formula unit across the simulation. The high c/a has "only" 0.85 pfu, however this is very high considering that the relaxed structure which has zero. Much of the difference arises from the more rapid breaking and remaking of bonds in the high c/a ratio case. These bonds typically last for, of order, 0.1ps, consistent with 300cm^{-1} lifetime broadening in Raman peaks. The different nature of the two structures is evident from the pictures, the high c/a looks more like alternating Ba and H layers, which the low c/a has hydrogen primarily in interstitial locations. S23

S10 Crystal Structures for BaH_4, I4/mmm low and high c/a (top); Cmcm-H2 and Cmcm-H3 (bottom) Bonds are shown for Ba-H less than 2.4Å, H-H less than 1Å. S24

S11 Density of states of $I4/mmm$ BaH_4 at 50GPa with 50% (red) and 100% (black) of H_2 units converted to H. The covalent states around -4 eV are entirely absent when all molecules are removed. The added H atoms contribute 2 states and 1 electron to the H^- bands, leading to metallic structures. S25
S12 Images showing the three C2/c structures found for BaH_4 in this work. The H^-, H_2 and H_3^- units are well defined in each case, leading to two negatively charged . S26
S13 ELF value at the $2b$ site (oct), $4d$ site (tet) and the ELF maxima on $4e$ sites ($\max_{oct}$) with the $2b$ site as middle point for the empty Ba lattices of low and high $I4/mmm$-BaH_4. . . . . S27
S14 a) ELF isosurface (ELF=0.32) for the pure $Pm\bar{3}n$ Ba sublattice of $BaH_{5.75}$ at 50 GPa (in yellow). Ba atoms represented as blue spheres and ELF maxima as green, pink and grey spheres. b) ELF isosurfaces, ELF=0.95 (in orange) and ELF=0.65 (in yellow) for $Pm\bar{3}n$ $BaH_{5.75}$ at 50 GPa. Ba atoms represented as blue spheres and the 3 non-equivalent H atoms as green, pink and grey spheres, respectively. S28
S15 ELF value at the middle H-H point for H-H distances up to 1.5 Å at 50 GPa. a) $BaH_{5.75}$, b) BaH_{12} S29
S16 Radial distribution functions from molecular dynamics simulations in $I4/mmm$ BaH_4 at various conditions. Each graph shows total g(r) normalised to total atomic density. S30
S17 **Equal-mass MD can be used to demonstrate melting : molten Ba** Details from the ”equal-mass” MD simulation in $BaH_{5.75}$ which melts at 1000K 100GPa a) Snapshot of the molten configuration (doubled cell, periodic boundary conditions suppressed). b) Mean squared displacements of the ”light” Ba (red) and Hydrogen (black, also x,y,z components showing homogeneous motion) S31

S18 **$BaH_{5.75}$ as $BaH_2(H_2)_x$** (left) Radial distribution function of the 100GPa/300K simulation, compared with those at 1000K in liquid and solid form. (right) Pre- and post-melting radial distribution functions, and the breakdown by species. Analysis of the bonding shows that the MD in typically 94% fully bonded, compared to the low symmetry, relaxed structure $BaH_2(H_2)_x$ (227 compared to the ideal 240/368 hydrogens in bonds). The lifetime of these bonds, as measured by counting changes in the bond list, is given in Table 3. . . . . S32

S19 The most stable found structure (Pc - $Ba_{16}H_{92}$) of $BaH_{5.75}$ relaxed at 50GPa (left) Showing 30 H_2 bonds pfu in range 0.813-0.936Å. (i.e. $BaH_2(H_2)_x$ - next shortest is 1.049Å. (right) Showing Ba-H bonds less than 3Å, illustrating that each hydrogen has precisely 4 Ba neighbours (i.e. is confined to a tetrahedron). S32

S20 Radial distribution functions from molecular dynamics simulations starting in the *Cmcm*H2 structure. Each graph shows total g(r) normalised to atomic density, and contributions from each bond type. S33

S21 Fourier transform of the velocity autocorrelation function from MD of the $BaH_{5.75}$ structure (100GPa/300K). The H_2 vibrations show a broad but unresolvable region between 2000 and 3000 wavenumbers. S36

S22 Radial distribution functions from molecular dynamics on BaH_{12}. Solid lines are total RDF, dashed lines count only the H-H distances. The sharp first peak at 0.8Å indicates the preferred molecular bondlength seen in these simulations and is almost independent of pressure. The compression with pressure is evident only in the second peak - the intermolecular distance. The second largest peak is the BaH nearest-neighbour separation, which can be seen to reduce with pressure. The overall crystal structure can be described as pseudocubic. S37

S23 Radial distribution functions at all pressures considered (300K) from molecular dynamics on BaH_{12}. S38

S24 **BaH_{12} as $BaH_2(H_2)_5$** Relaxed P1 BaH_{12} structures at 50, 100 and 150GPa (left to right) Note the 100GPa case two Ba atoms have moved across the periodic boundary conditions. Analysis of molecular dynamics simulations from this structure reveals that the number of ”H_2 bonds” (criterion: atoms with one neighbour within 1Å) is 5 per formula unit. This conclusion is remarkably robust to reasonable choice of the bondlength. By contrast, the more-easily calculated bond criterion ”number of H-H distances less than 1Å ” is very sensitive to the choice of cutoff. This is because there is a significant proton exchange via the H_2+H^- →H_3^- →H^-+H_2 mechanism. S39

List of Tables

S1 Bader charges and ELF basin populations on the metal atoms (M), hydrogens on 4*e* sites (forming hydrogen pairs) and hydrogens on 4*d* sites (tetrahedral sites) for the $I4/mmm$ structures at 50 GPa S20

S2 Bader charges and ELF basin populations for the *Cmcm*-H3 structure at 50 GPa (a=3.462 Å. b=7.740 Å, c= 5.080 Å). S28

S3 Details of molecular dynamics simulations. WP is the Weaire-Phelan structure, Mass refers to the cation, bonds is the average number of hydrogen atoms within 1Å of precisely (one,two) other atoms. Broke is the number of times and atoms move into or out of the 1Å range of another, and this is normalise with time and number of bonds to give a number of such "events" per bond per ps. Relating this to the lifetime of the bond is complicated. e.g. harmonic bond oscillating beyond 1Å will record four "events" (two atoms, making and breaking) without being a genuine bond breaking. The common proton exchange process also records four "events" (one atom makes a bond, one breaks a bond, the central atom both makes and breaks). However in most of the recorded H_3 creation events, the "reaction" is "unsuccessful" and the initially-atomic hydrogen leaves as an atomic hydrogen. Taken together, a rough estimate is that bond breaking occurs with a frequency one order of magnitude less than the "Event" rate. Bondlength is the position of the first peak in the radial distribution function. S43

S1. How to define an H-H covalent bond?

There are many measures which can be used to define a covalent bond in an *ab initio* molecular dynamics simulation. A good measure should be a parameter which can distinguish between bonded and unbonded pairs.

S1.1 Bondlength

The easiest approach is to define some cutoff distance R_{bond}, and declare that any pair of hydrogen atoms closer than this are ”bonded”. This can be immediately evaluated from integrating the radial distribution function $g(r)$.

For perfect crystals $g(r)$ is zero between shells of neighbours. In many cases of md, including solid hydrogen, there of a large range where $g(r) = 0$ and the hydrogen bonded pairs can be unambiguously identified, even though the choice of R_{bond} is ambiguous, because any cutoff with $g(R_{bond}) = 0$ will give the same results.

In the present work, $g(r)$ does not go to zero. This however there is often a distinct minimum at around 1 Å. In the static structures we invariably finds that $g(r = 1\text{ Å}) = 0$. $R_{bond} = 1$ Å, therefore a pragmatic choice which we adopt as part of the definition. However, one must be careful that any measure of bonding is not highly sensitive to this choice.

The second ingredient of defining bonds from MD comes from investigating ”Bond-breaking” events. In practice we see two types of event.

- A sensible definition requires R_{bond} to be large enough that an oscillating molecule is not marked as ”breaking” if the bondlength briefly extends beyond R_{bond} at its largest extent. This is why our R_{bond} must be set larger than one might expect a H-H bond to be.

- Real bond breaking events in the current system come from reactions between atomic and molecular hydrogen. These involve an H^- approaching an H_2 molecule, forming an intermediate state of an H_3^+ unit, which then breaks so that the ion is now part of a molecule, and one atom of the molecule is an ion. We write this process as

$H^- + H_2 \rightarrow H_3^- \rightarrow H_2 + H^-$

where the ordering of the hydrogen atoms in the formula is assumed unchanged. In isolation, this could be regarded as a double-well potential, but in the condensed phase each molecule as many option for rebonding.

Our $R_{bond} = 1$Å criterion is long enough to flag the H_3^- unit as two bonds, and so we observe that the "number of bonds" increases during these reactions in a way that is very sensitive to the choice of R_{bond}. Thus we refine the measure by counting the number of atoms with precisely one neighbour within 1Å , and dividing by 2 to get the number of bonds. One can readily verify that during the processes described above this measure give one covalent bond throughout. It turns out that this measure is insensitive to choice of R_{bond}.

S1.2 Topological analysis of the electron density and ELF

There have been different approaches to analyze the chemical bonding in crystals. Probably, one of the most straightforward and mathematically rigorous is the Quantum Theory of Atoms in Molecules developed by Bader.[1] It is based on the topology of the electron density. The topological analysis first identifies the critical points of the scalar fields, i.e, points where their gradient vanishes. These are then classified as maxima, first-order saddle points, second-order saddle points, or minima. The maxima of the electron density are usually located on the atoms, whereas the first-order saddle points are associated with chemical bonds (denoted bond critical points (b.c.p.)). The electron density and the Laplacian at these points are used to characterize the bond strength and its character. For instance, in a molecule such as H_2 the b.c.p. is characterized by high electron density and negative laplacian (electrons are locally concentrated). The crystalline space can be also partitioned in topological atoms defined by the union of the electron density maxima with their attraction basins and delimited by surfaces obeying the zero-flux condition for the electron density. Their charge can be calculated from the integration of the electron density within these regions. These regions are non-overlapping and additive, recovering the total volume of the crystal.

The electron localization function was introduced by Becke and Edgecombe in 1990 for the

analysis in real space of the electron localization,[2] and later reinterpreted by Savin in terms of the Pauli kinetic energy density (t_p).[3] By definition, ELF is a relative measure of the electron localization with respect to the homogeneous electron gas (HEG). In general, the ELF value approaches 1 in regions of the space where electron pairing occurs (e.g., atomic shells, bonds and lone pairs). In analogy with QTAIM, a partition of the space based on the ELF can be performed. It consists of non-overlapping basins with well-defined chemical interpretation (cores, bonds, lone pairs). Moreover, the basin charges come from integration of the electron density within these regions. For example, a typical molecule will feature an ELF maxima in the middle of the interatomic distance associated with the bond. What is more, a region of the space (ELF maxima basin) can be attributed to it, with an electron population obtained by integration of the electron density in that region. Hydrogen constitutes an exception. A maximum does not appear along the H-H bond since the hydrogen molecule only contains two electrons. In this case, a high ELF isosurface encapsulates both atoms. Typically, a H_2 molecule is predicted to exist if the hydrogen atoms are connected at a value of ELF higher than 0.85, i.e, if the minimum ELF value in between the hydrogen atoms is above 0.85.

S1.3 Wavefunctions

A quantum wavefunction can be expanded in any complete set of orthogonal basis functions. In a DFT calculation we use a plane wave basis set so there are no molecular orbitals. Plane waves offer the big advantage that one can systematically approach a complete set by increasing the cutoff, and they are automatically orthogonal. A basis set of localised orbitals is typically non-orthogonal and therefore simultaneously overcomplete and undercomplete.

To use the Kohn-Sham wavefunctions to identify molecules one must project them from plane waves onto localised basis functions. This can be done using maximally localised Wannier functions. Sup. Figs S1, and S2 show the projection of the Kohn-Sham bands identified as bonds onto maximally localised Wannier functions, the localisation in real space and the identification in reciprocal space are clear.

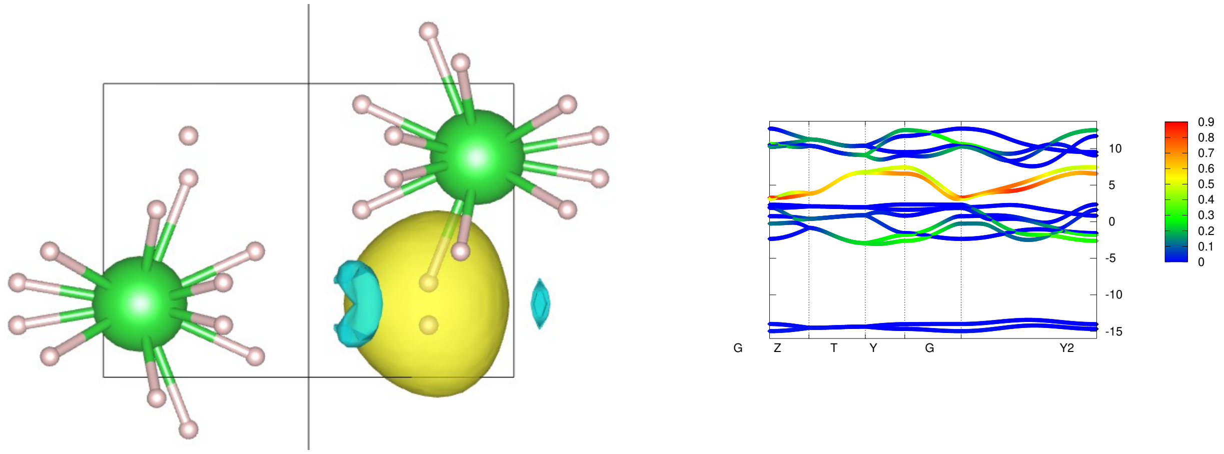

Figure S1: (right) Projection of Kohn-Sham band onto Maximally Localised Wannier function for BaH_4 $I4/mmm$ structure showing localisation on a single H_2 unit. (right) Bandstructure of BaH_4 with colours showing projection onto H_2 covalent bond orbitals.

Some 20% spillage of the low-lying Ba 6s states projected onto the Ba-p orbitals give some indication of the ambiguity in using the projection: it is good for qualitative measure. This identified localised states on the Ba and the H^+ as one expects for ionic materials (Sup. Fig.S2c-d).

The H_2 unit can be considered in terms of its two molecular orbitals, the bonding $1\sigma_g$ corresponds to the band some 5eV below the Fermi energy, while the antibonding $1\sigma_u$ state is above the Fermi energy. The H_3 unit can also be considered in terms of molecular orbitals. Since it has three atoms, H_3 has precisely three molecular *s*-orbitals, most simply written[1] as $1\sigma_g$, $1\sigma_u$ and $2\sigma_g$, whose wavefunctions have zero, one and three nodes. H_3^- implies that $1\sigma_g$ and $1\sigma_u$ are occupied.

The Wannier approach was also applied to the Cmcm-H3 structure, which has H_3^- units (Fig.S2). The H_3 $1\sigma_g$, $1\sigma_u$ electronic states are located in the same region of space. This enables the MLWF to generate combination of bonding and nonbonding states localised on one end of the H_3 unit. Such a state is preferred by the MLWF procedure because it is more localised than either $1\sigma_g$ or $1\sigma_u$ states which are spread across three atoms. Projection of this MLWF state onto bandstructure shows it occupies both the 5eV region where the H_2 bands lie in other compounds, and the DoS peak just below the Fermi Energy degenerate with he H^- states (Sup. Fig.S2e-f). We propose that the in the molecule orbital picture $1\sigma_g$ and $1\sigma_u$ can be assigned to these energy regions, while $2\sigma_u$

[1] Sometimes the three names ”bonding, nonbonding and antibonding” are applied to these states.

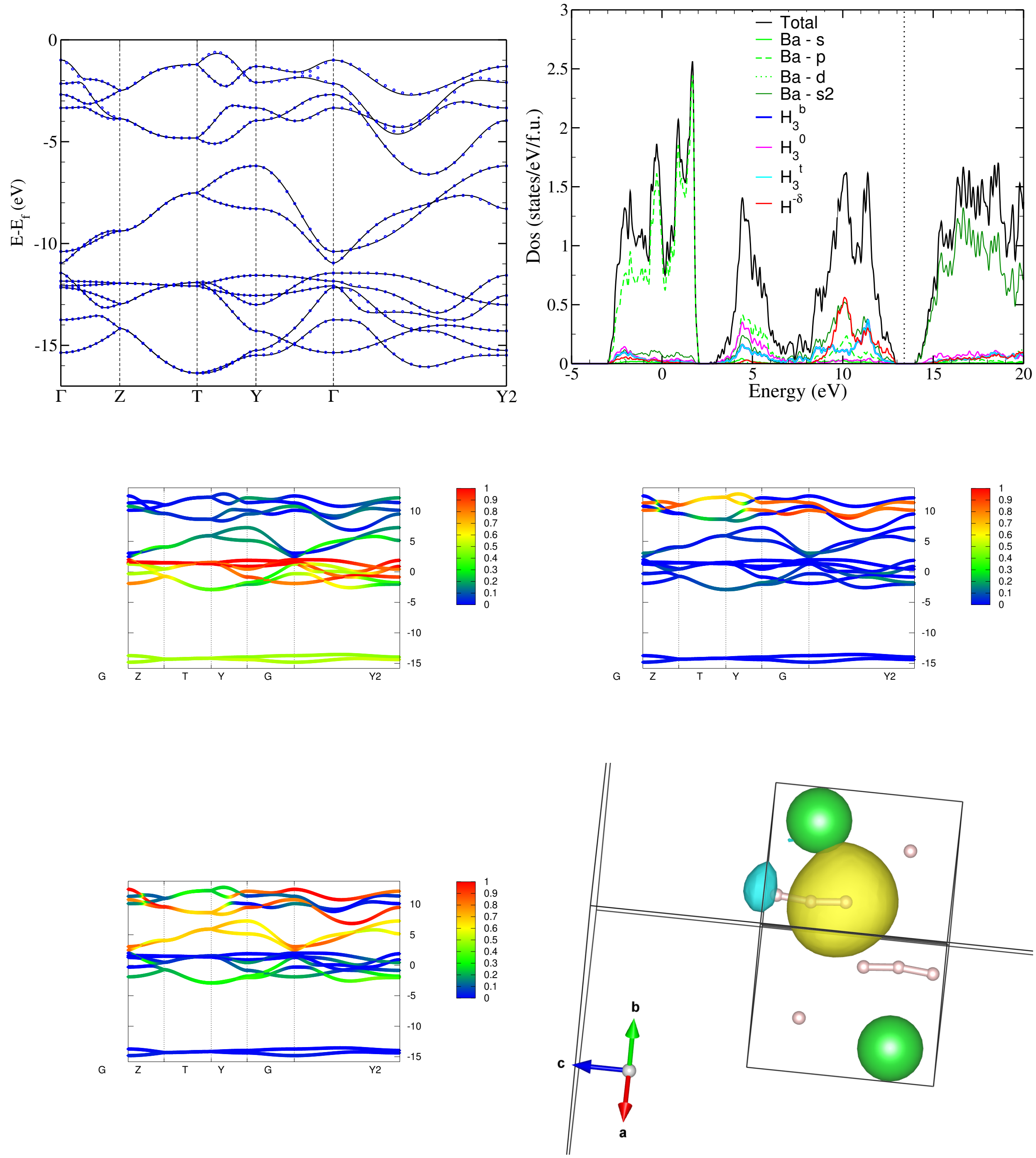

Figure S2: (right) Projection of Kohn-Sham band onto Maximally Localised Wannier function for the *Cmcm*-H3 structure. (a) Band structure: bands are enumerated from lowest energy upward, including the Ba 6s (b) Projection of MLWF onto Bloch states. (c) Projection onto Ba 5p MLWF states. (d) Projection onto atomic H 1s MLWF states (e) Projection onto MLWF states for H_3^- (f) Isosurface of an H_3 MLWF

lies above the Fermi energy.

S2 Density functional and ab initio molecular dynamics calculations

S2.1 Codes and settings

For static relaxations we carried out density functional theory calculations using the CASTEP code, with ultrasoft pseudopotentials and the PBE functional.[4,5] We used a plane-wave basis with a 700 eV cutoff and a k-point spacing better than 0.04 $Å^{-1}$. Key results were repeated using LDA and BLYP functionals,[6–9] which give similar qualitative results but have some minor effect on transition pressures. Molecular dynamics were run with a a cutoff energy of 463 eV and k-point grids up to 26^3 in CASTEP, with a timestep of 0.5fs in the NPT ensemble, and were followed by geometry optimisations of snapshots to zero temperature.

ELF and Bader analyses were carried out using the CRITIC2 program,[10] and applied to all single unit cell static structures

Wannier analysis was carried out using the wannier90 code[11]

S2.2 Fictitious Mass Molecular Dynamics

The partition function in the NPT ensemble:

$$Z = \sum_i \exp(-H_i/kT) = \sum_i \exp(-U_i/kT)\exp(-PV_i/kT)\exp(-KE/kT)$$

Where U_i is the potential energy of the microstate, V_i is the volume and KE is the Kinetic energy. In the classical limit, the kinetic energy is proportional to temperature, so the final term factors out. We observe that the first two terms are independent of the atomic masses, so we are free to choose them as we wish.

In the case of barium hydride, the two orders of magnitude difference in mass means that in conventional MD the bariums would scarcely move. However, the fictitious mass approach enables us to correctly sample the partition function, so phase transformations involving the Ba

atoms can be observed. Likewise a simulation run above the melting temperature will move into the phase-space region of the melt. The partition function is correctly sampled, so equilibrium thermodynamic properties are correct. However the dynamics of the melting process, or the vibrational spectra, will not be correct.

The most challenging thing to determine in this partition function is the definition of i - this indicates ”Microstates corresponding to the I4mmm structure”. Of course, finite temperature microstates have no symmetry, and NPT simulation in designed to allow for a phase change. In practice, we monitor the box dimensions and the fraction of molecules to determine distinct phases.

We derived this method independently, but it seems such an obvious idea that we assume it is not new.

S2.3 Hydrogen in jellium

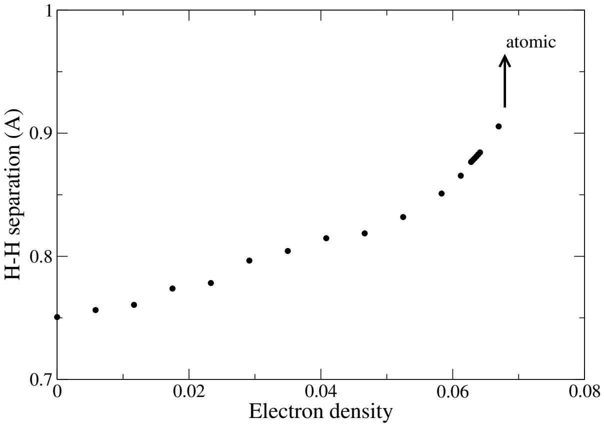

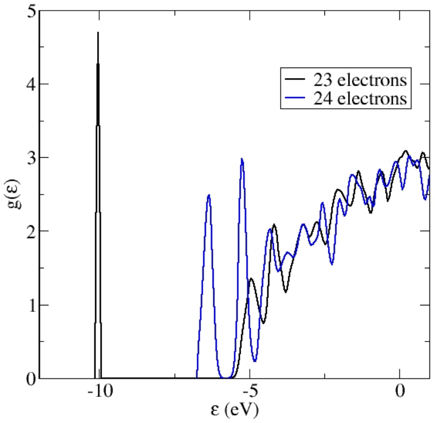

Figure S3: **The maximum electron density into which an H_2 molecule can be embedded is 0.07 Å $^{-3}$.** a) Plot of bondlength vs DFT Calculation using PBE hydrogen embedded in a jellium background at the shown electron density. Calculations were done with one hydrogen molecule in a 343 Å ^{3} box with a 16^3 k–point grid. Similar calculations using BLYP give the bond breaking at the same density b) Bandstructure calculations (density of states) for the system described above with a total of 23 or 24 electrons in the system. The covalent bond in the 23 electron system lies some 10 eV below the Fermi energy, or 4.5 eV below the bottom of the free electron band. After bond-breaking, the atomic states lie at the bottom of the free electron band. Other oscillations in the free-electron band structure come from the cubic symmetry of the supercell and are not relevant.

Breaking the H_2 bond can be due to physical confinement in an interstial site: ”chemical pressure”, or by electronic effects ”antibonding”.

The embedded atom method is one of the most enduring and successful techniques in materials modelling. It was introduced with the hypothesis that the binding energy of hydrogen in a metal would depend on the electron density of the other atoms, and there was an optimal value for this. Hence the electronic contribution to bond breaking can be calculated in isolation by embedding the H_2 molecule in a homogeneous electron gas. This system has been studied in detail,[12] and here we repeat that work with the methods employed in this paper. For low electron density the band structure comprises a free electron-like density of states which lies much higher in energy than a clearly-defined H_2 bond. Above 0.06 s e Å^{-3}: the bond breaks spontaneously, evidenced by a discontinuous jump in the H-H separation, the vanishing of the flat Kohn-Sham band defining the molecule and the appearance of an atomic-hydrogen state at the bottom of the free-electron band (Sup. Sup. Fig. S3). This sets an upper limit on where molecular hydrogen can be found.

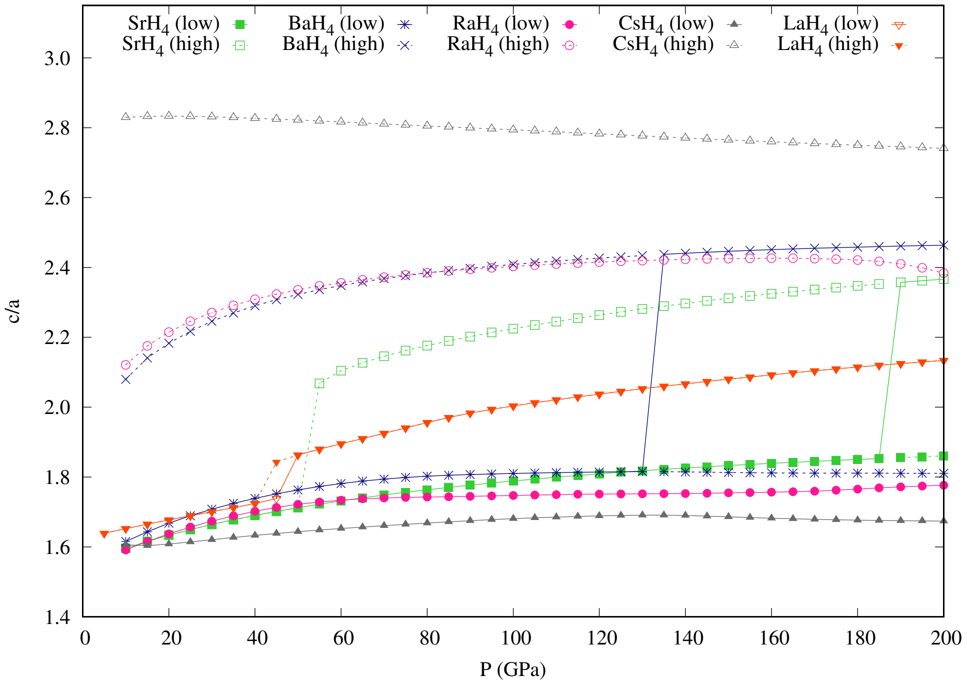

Figure S4: Evolution of c/a with pressure for the $I4/mmm$ structures. Solid and open symbols correspond to the low and high c/a structures. The $I4/mmm$ structures with the lowest enthalpies at each pressure are indicated by solid lines.

S3 $I4/mmm$ structures

S3.1 Comparing I4/mmm materials

We considered $I4/mmm$ structures for SrH_4, LaH_4, CsH_4 and RaH_4 to determine trends across the periodic table making no definitive claim about the stability of the calculated compounds. c/a ratio, lattice parameters and ELF-basin populations are shown in Sup. Figs. S4, S5 and S6.

BaH_4 is discussed in detail in the main text. It is unusual in that the low c/a structure calculated to be stable in DFT is different from the high c/a ratio structure seen in the experiment. Nevertheless, both structures are local energy minima. Many other materials exhibit the same theoretical coexistence of two energy minima in the same I4/mmm structure.

SrH_4: Strontium is above Barium on Group 2A, and calculations show the atomic and molecular forms do adopt separate enthalpy minima, with the atomic form only becoming stable above 185GPa. Experiments on SrH_4 have not reached these pressures. The molecular form is non-

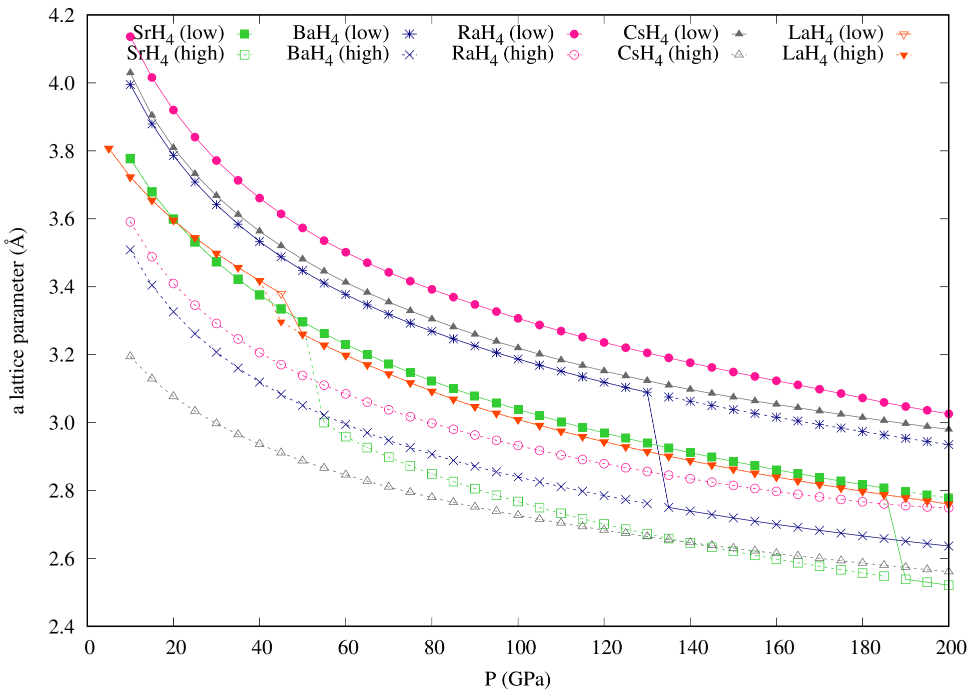

Figure S5: Evolution of a lattice parameter with pressure for the $I4/mmm$ structures. Solid and open symbols correspond to the low and high c/a structures. The $I4/mmm$ structures with the lowest enthalpies at each pressure are indicated by solid lines.

metallic, e.g. at 50GPa there is a bandgap of about 2eV (Sup. Fig.S7)

CsH_4: Caesium lies beside Barium, and calculations show the atomic and molecular forms do adopt separate enthalpy minima, but the atomic form is unstable up to 200GPa. This is despite the fact that, being monovalent, the material is calculated to be metallic with the Fermi level in the atomic hydrogen band. Being so chemically unfavoured suggests that this structure will not be stable, probably decomposing into CsH[13] and hydrogen or a polyhydride.

RaH_4: Radium lies below Barium in the periodic table, and is highly radioactive so the nature of its high-pressure hydrides is unlikely ever to be tested. Calculations suggest that at all pressures up to at least 200GPa the low c/a molecular structure is stable. The band structure is similar to BaH_4 with somewhat broader bands and a DFT/PBE band gap which closes at 50GPa,

LaH_4: Lanthanum is next to Ba in the periodic table, and typically forms trivalent ions. Previous work[14] studied the high-pressure form of LaH_4. The pressure dependence of the bond in the LaH_4 shows a trend towards steady lengthening with pressure, with no distinct high and low

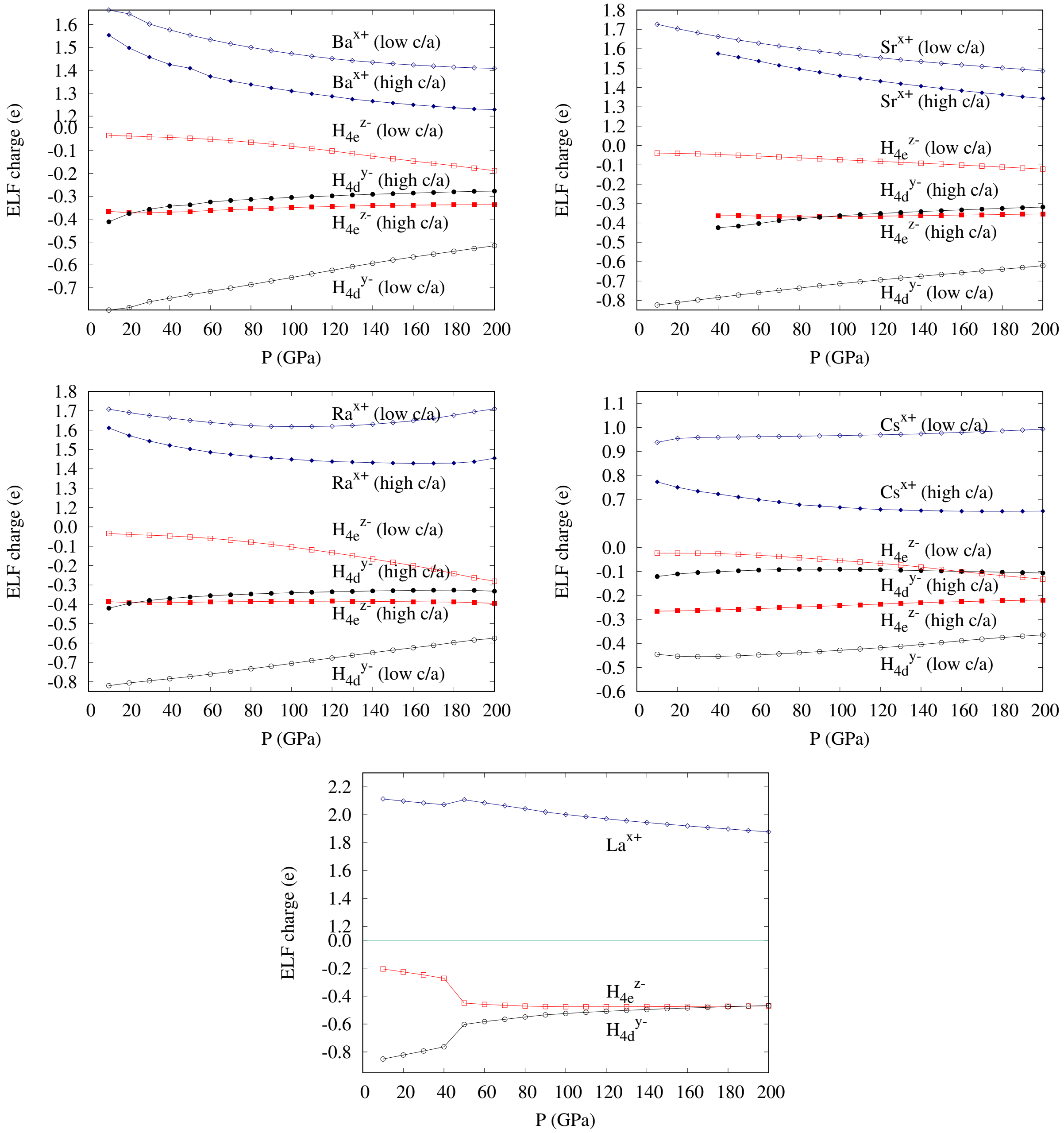

Figure S6: a) Charges as a function of pressure in $I4/mmm$ compounds based on the ELF topology for the metal and hydrogen atoms located on 4*e* and 4*d* sites

variants. At 50GPa two short H-H separations are already 1.5Å apart in the density of states, and the characteristic “molecular” peak has vanished (Sup. Fig.S7) The LaH_4 is always metallic, even in the molecular case, with the Fermi level lying in the La *sd* conduction band. Given this, it is likely that LaH_4 is unstable with respect to higher polyhydrides.

The band structures reveal significant similarities between the different materials , and differences between atomic and molecular forms (see Density of states at 50GPa). For BaH_4, we calculate five distinct groups of state: low-lying Ba 5s (-22eV) and 5p orbitals (-10eV). Then at -4eV come the H_2 (0.79Å) molecular bonding orbitals. Closest to the Fermi energy are the atomic hydrogen H^- hybridized with Ba 5d: population analysis in atomic basis sets suggests that the Ba 6s are unoccupied.

In the Sr compound, this allocation is even clearer. In Ra, the electronic bands are broader but energy gaps in the density of state mean there is no hybridization. At 50GPa the H_2 bond in both Sr and Ra is 0.79(1)Å

Overall, the comparison of the adjacent materials indicates that Ba is unusual in showing the atomic-molecular transformation at the lowest pressure. The band structures are remarkably similar across all materials, with the position of the Fermi energy determined by the cation valence. We have not done extensive structure searching: our molecular dynamics simulations indicate that the molecular hydrogens are orientationally disordered at room temperature, while quantum nuclear effects are likely to be important at zero temperature. Given the emerging picture that these materials can be thought of as dihydrides with interstitial H_2 it is unlikely that the metallic compounds with monovalent and trivalent cations are stable.

Table S1: Bader charges and ELF basin populations on the metal atoms (M), hydrogens on 4*e* sites (forming hydrogen pairs) and hydrogens on 4*d* sites (tetrahedral sites) for the $I4/mmm$ structures at 50 GPa

System	Bader charge			ELF basin population		
	on M (e)	on H_o (e)	on H_t (e)	on M (e)	on H_o (e)	on H_t (e)
$I4/mmm$-BaH_4 (low)	+1.174	-0.041	-0.546	8.446	1.047	1.731
$I4/mmm$-BaH_4 (high)	+1.036	-0.253	-0.265	8.591	1.367	1.337
$I4/mmm$-SrH_4 (low)	+1.247	-0.049	-0.574	8.355	1.050	1.772
$I4/mmm$-SrH_4 (high)	+1.143	-0.250	-0.321	8.444	1.362	1.416
$I4/mmm$-RaH_4 (low)	+1.278	-0.047	-0.591	8.350	1.052	1.773
$I4/mmm$-RaH_4 (high)	+1.136	-0.277	-0.291	8.497	1.390	1.362
$I4/mmm$-CsH_4 (low)	+0.691	-0.031	-0.315	8.040	1.029	1.451
$I4/mmm$-CsH_4 (high)	+0.538	-0.194	-0.075	8.290	1.258	1.098
$I4/mmm$-LaH_4	+1.536	-0.322	-0.446	8.893	1.604	1.450

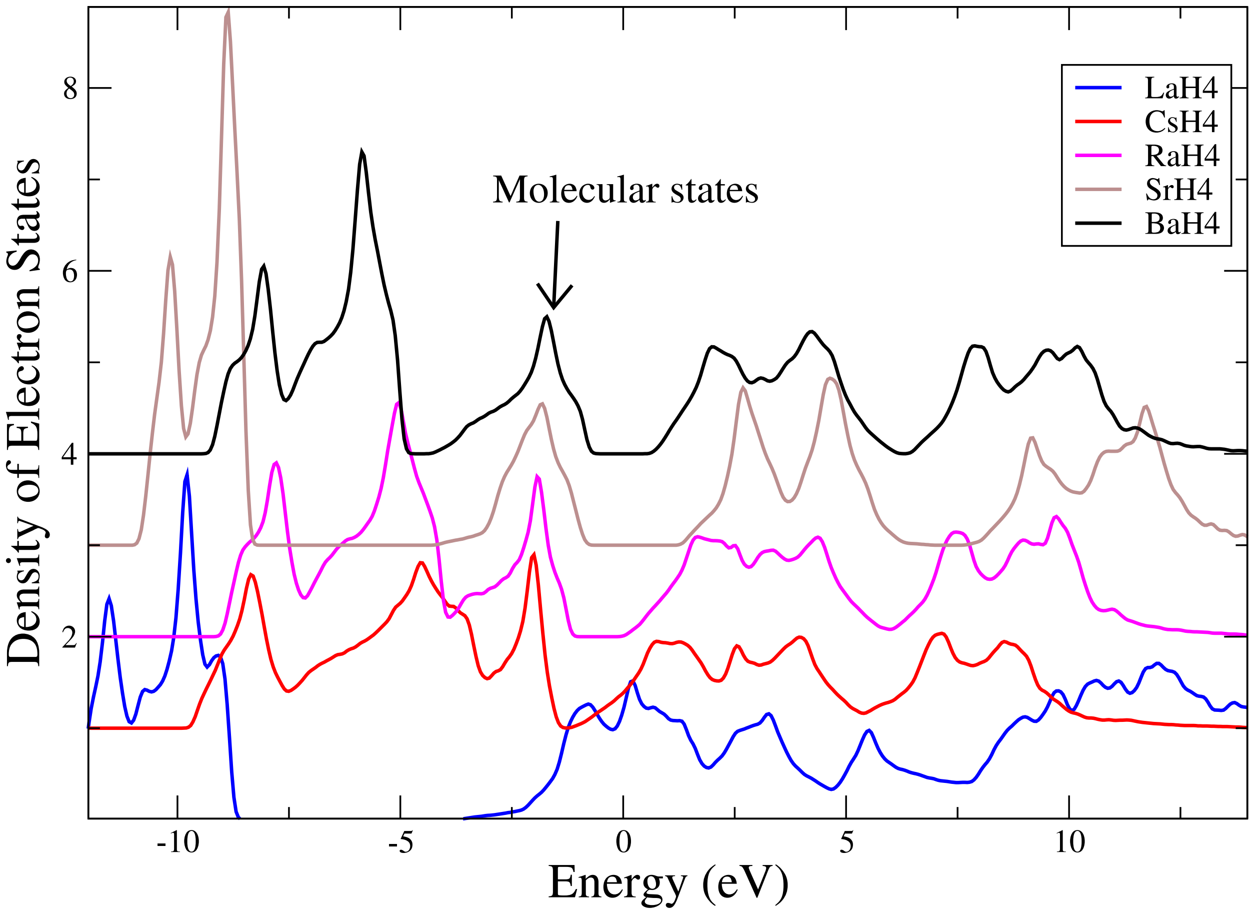

Figure S7: Band structures at 50GPa in SrH_4, BaH_4, LaH_4, CsH_4 and RaH_4. The zero of energy has been offset to place the peak of the H_2 ”band” together, as indicated by the arrow. The calculated peaks contain 2 (not shown), 6 (double peak, widely separated e.g. in Cs), 2 and 4 (double peak) electrons per formula unit respectively. In BaH_4 these can be interpreted chemically as Ba 5s, Ba 5p, molecular $H_2\sigma$, and atomic H 1s. The Fermi energy lies in the gap at +6 eV and the conduction band has 5d and 6s character.

S3.2 Can the atomic and molecular forms coexist in BaH_4?

When two different forms of hydrogen can be found in a single structure, it opens the question of whether the real material should contain a mixture of each. This could naively be treated as a mean field two-state system, with the probability of finding a given state being $e^{-\Delta G/kT}/[1+e^{-\Delta G/kT}]$ with ΔG being the free energy difference. At room temperature this would be close to 50%, and no discontinuous phase transformation could be observed.

However, the strong coupling between the atomic/molecular transition and the strain suggests that this view is overly naive. Stabilization of the atomic form occurs only in concert with a large

enlongation of the octahedral interstice, which is incompatible with a molecular form in an adjacent site. A more appropriate model is that of Bragg and Williams (BW) which explicitly includes an interaction enthalpy between adjacent sites. BW considered the interaction to be mainly a short-ranged bonding effect, however here it seems that strain provides the major contribution.

To test this we ran a series of molecular dynamics type calculations on a number of cells. The purpose of these was to sample the available phase space, rather than study dynamical trajectories. The partition function is independent of the atomic masses, we set the H_2 and Ba masses to be equal. The results were monitored by tracking all dihydrogen pairs, defined geometrically as atoms less than 1Å apart. We record each occasion when these connections change from one timestep to the next as an indication of how often the bonds break. We also calculated the radial distribution functions, the mean squared displacements, by visualisation of the trajectories using vmd, and by relaxation of selected snapshots.

Long MD runs of single unit cells, just 10 atoms, followed by static relaxation was used to track dynamic instability against Γ-point phonons, it was found that starting from the low-c/a:

- Molecular I4/mmm 50GPa with 300K rotated molecules and rebonded, relaxation gives structures with H_3^- units.

- Molecular I4/mmm 50GPa with 600K transformed to a Cmcm-H3-like structure by breaking and reforming bonds rather than rotating molecules.

- Molecular I4/mmm 200GPa 300K transforms to the atomic phase, with molecules diffusing in the plane by a bond-making and breaking mechanism.

These small simulations with unphysical masses allow us to span the phase space. We also ran larger cells with 80 atoms and correct masses (137:1) to investigate the dynamics. We ran NPT simulations with a Parrinello-Rahman barostat which allows the cell to fluctuate but only permits small relative movements of the Ba atoms. At 50GPa MD shows that the hydrogen molecules retain their identity and rotate rather than remain orientated along the *c* axis. A consequence of that is further reduction in c/a so that the mean lattice parameters are a=3.6 (3.38), b=3.8 (3.38)

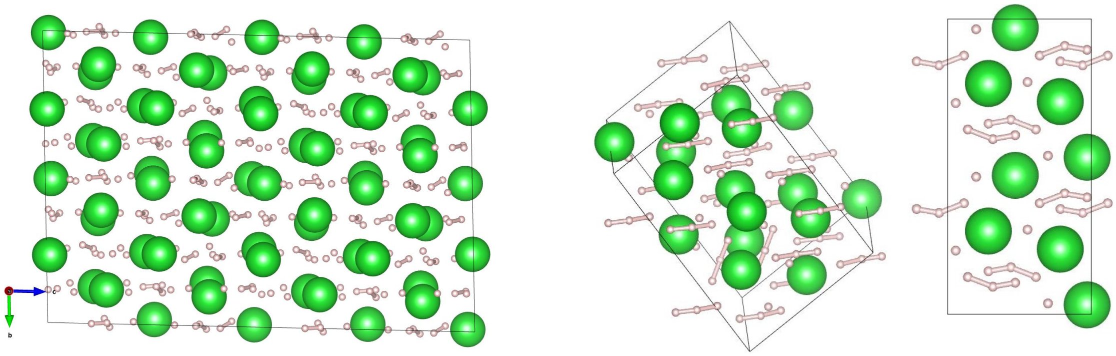

Figure S8: Structures from $I4/mmm$ MD simulations. (left: MD snapshot 50GPa/300K; centre: snapshot after relaxation with H_3^- units; right: 200GPa snapshot after relaxation with H_2 units)

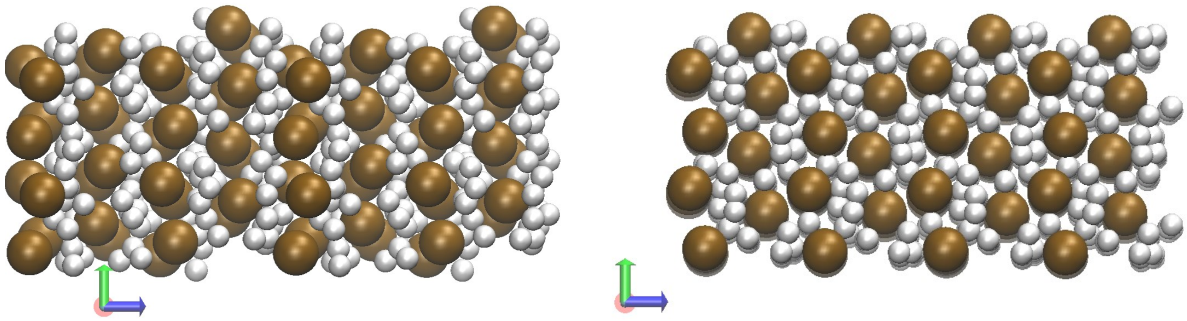

Figure S9: **At room temperature both high and low c/a structures of I4/mmm BaH_4 contain molecules** Snapshots from PBE MD simulation of (a) high $< a >= 5.38, < b >= 5.40, < c >=$ 12.29 and (b) low MD simulations at 200GPa and 300K $< a >= 5.807, < b >= 5.50, < c >= 11.2$. Analysis of the number of dihydrogen pairs at less than 1Å shows a distinct difference. The low c/a version averages 0.99 molecules per formula unit across the simulation. The high c/a has "only" 0.85 pfu, however this is very high considering that the relaxed structure which has zero. Much of the difference arises from the more rapid breaking and remaking of bonds in the high c/a ratio case. These bonds typically last for, of order, 0.1ps, consistent with 300cm^{-1} lifetime broadening in Raman peaks. The different nature of the two structures is evident from the pictures, the high c/a looks more like alternating Ba and H layers, which the low c/a has hydrogen primarily in interstitial locations.

c=5.2 (6.07), with 0K values in brackets. At 600K a similar scenario plays out, with molecules breaking and reforming on a sub-picosecond timescale.

Constant features across all MD on BaH_4 are the creation of close to one H_2 pair per formula unit, and the non-metallic bandstructure of the relaxed structures.

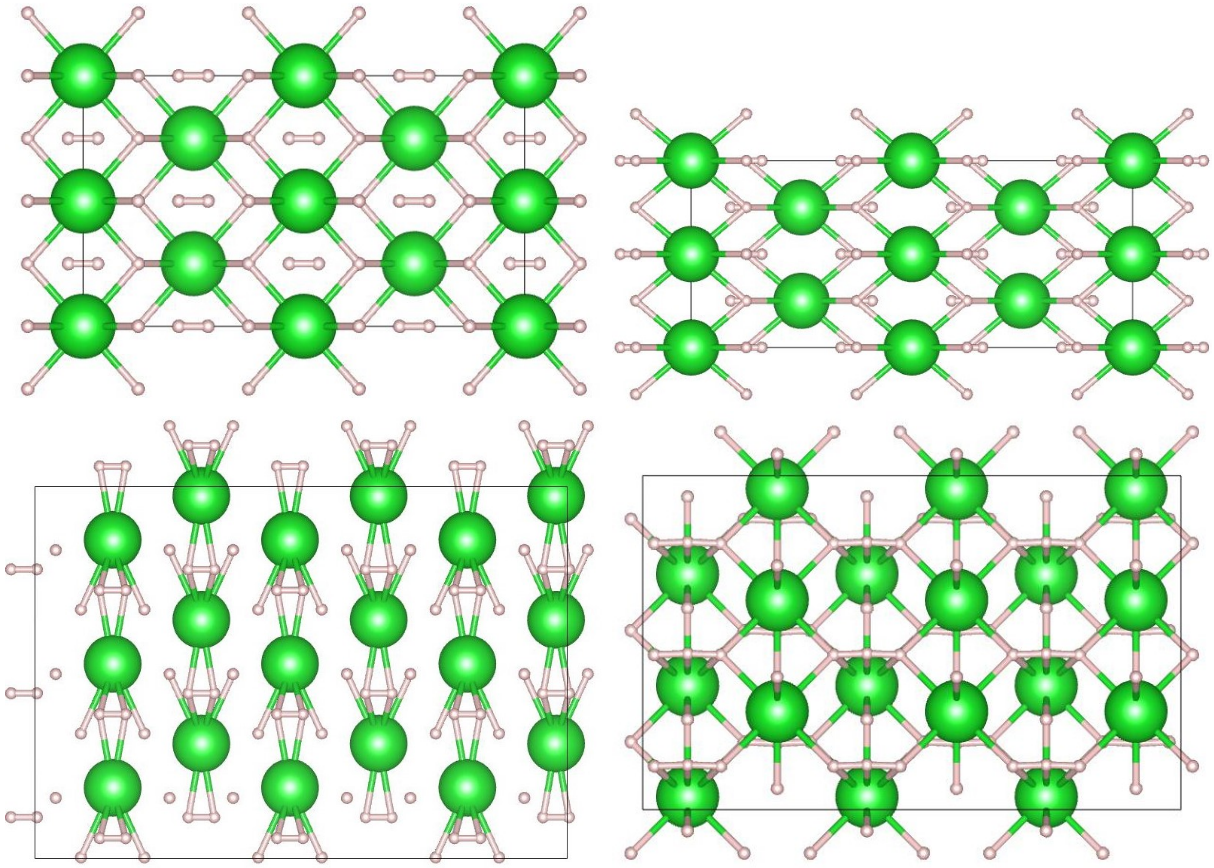

Figure S10: Crystal Structures for BaH_4, I4/mmm low and high c/a (top); Cmcm-H2 and Cmcm-H3 (bottom) Bonds are shown for Ba-H less than 2.4Å, H-H less than 1Å.

S3.3 Stability of $I4/mmm$ BaH_4

Molecular dynamics runs (Sup. Figs. S8 and S9) show that the hydrogens in I4/mmm are dynamically disordered and only obey I4/mmm symmetry on average. Moreover, geometry optimisation from molecular dynamics runs finds different symmetries, including the Cmcm-H3 which appears to be to low temperature ground state (Sup. Fig.S10).

S3.4 Substoichiometric $I4/mmm$ materials

In experiments, BaH_4 is formed from BaH_2 in a hydrogen-rich environment. We therefore investigated substoichiometric versions of the $I4/mmm$ structure, replacing the H_2 unit with a single H molecule.

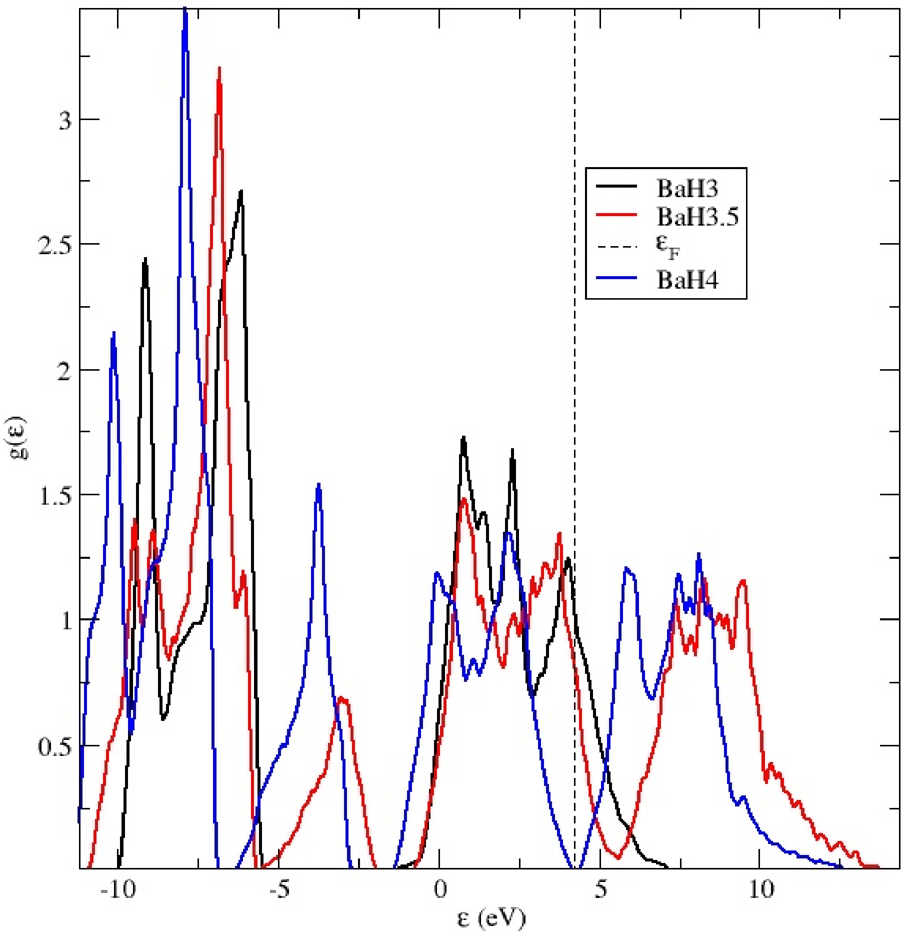

Figure S11: Density of states of $I4/mmm$ BaH_4 at 50GPa with 50% (red) and 100% (black) of H_2 units converted to H. The covalent states around -4 eV are entirely absent when all molecules are removed. The added H atoms contribute 2 states and 1 electron to the H^- bands, leading to metallic structures.

With all H_2 units changed to H, the stoichiometry is BaH_3 and the c/a ratio relaxed to $\sqrt{2}$. the hydrogens remain in the high symmetry tetrahedral and octahedral sites, such that the overall symmetry of this structure is Fm-3m and the Ba are located on the sites of an fcc lattice. With 50% of H_2 changed to H, we find the c/a at 1.6.

The band structure of these off-stoichiometric structures have a clear signature of the disappearance of the H_2 bands and a broadening of the H^- band leading to closure of the bandgap (Sup. Fig.S11).

Nevertheless the fcc all-atomic BaH_3 structure in DFT is highly unstable to molecule formation

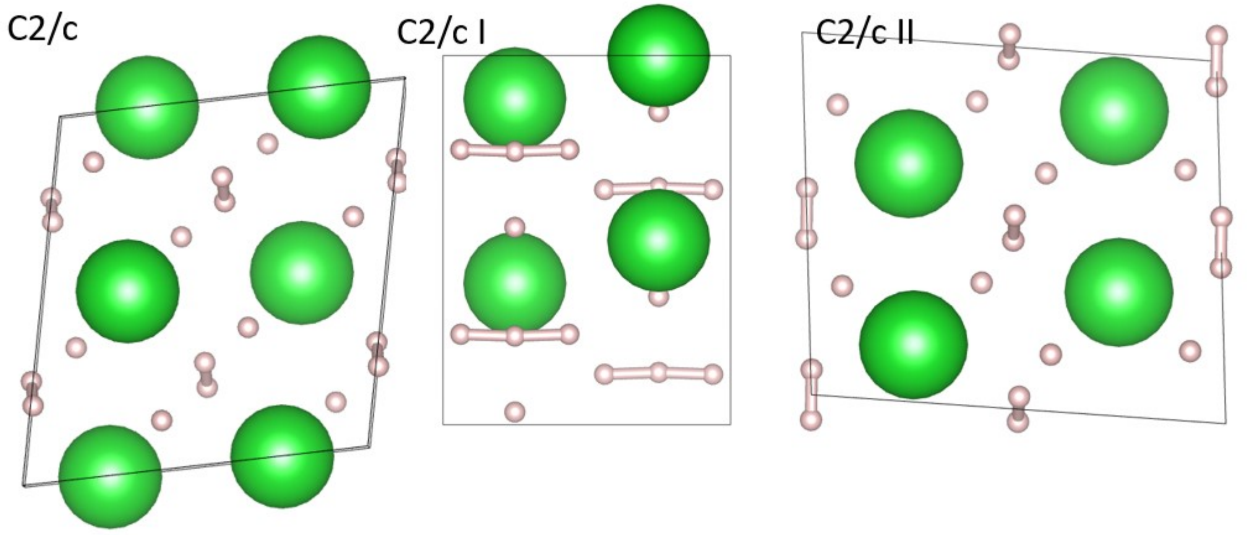

Figure S12: Images showing the three C2/c structures found for BaH_4 in this work. The H^-, H_2 and H_3^- units are well defined in each case, leading to two negatively charged

- by about 0.7eV per formula unit, whether one compares decomposition into cotunnite BaH_2 and BaH_4, or a BaH_3 structure with one molecule and one empty octahedral site.

In molecular dynamics simulation with a single ”missing” H we see that the hydrogen can diffuse on a sub-picosecond timescale. The mechanism for this diffusion is the transfer of a hydrogen atom to molecule forming an H_3^- complex, with one of the original pair moving away to become a free atom, leaving a new pairing. We observed this migration mechanism by inspection in vmd and by monitoring the graph of connections $< 1Å$ between molecules in the MD.

S4 Empty lattices

We studied the ELF of the Ba sublattices of the $I4/mmm$-BaH_4 structures at a range of pressures (Sup. Figs.S13). These structures are typically unstable and metallic. For the low c/a structures ELF maxima are centered on tetrahedral and octahedral sites. For the high c/a structures, there are ELF maxima on tetahedral sites, with similar ELF value to that ones of the low c/a structures, but two different ELF maxima with considerably higher ELF value than that at the octahedral site appear. H atoms and H_2 molecules locate on this ELF maxima in the full compounds.

It demonstrates that the ELF maxima in the empty lattice are good predictors of the position of the hydrogens in the hydride. Electrons which are already partly localized can lower their energy by forming H^- ions.

In analogy to it, the position of the ELF maxima of the Ba sublattice of high symmetry $BaH_{5.75}$ is the same than that of the hydrogens in the hydride compound (See Fig. S14).

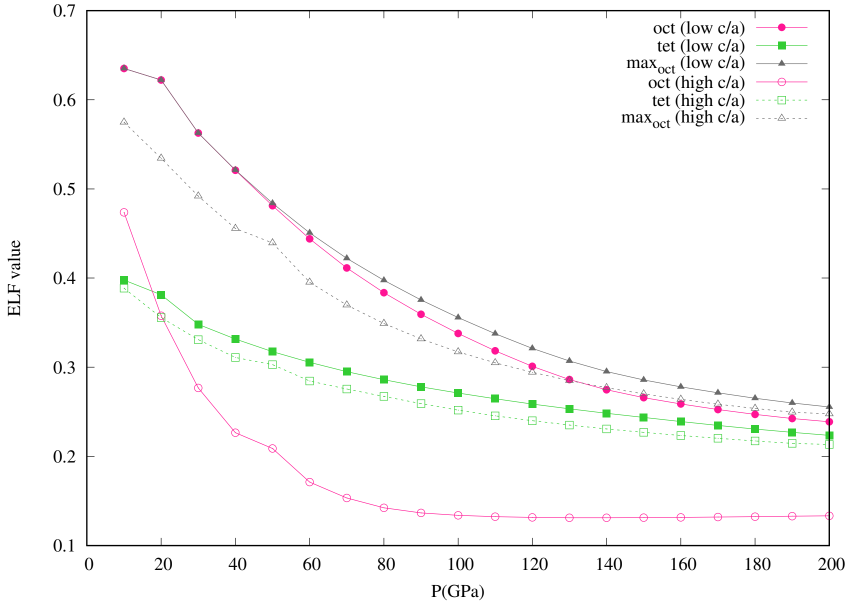

Figure S13: ELF value at the $2b$ site (oct), $4d$ site (tet) and the ELF maxima on $4e$ sites ($\max_{oct}$) with the $2b$ site as middle point for the empty Ba lattices of low and high $I4/mmm$-BaH_4.

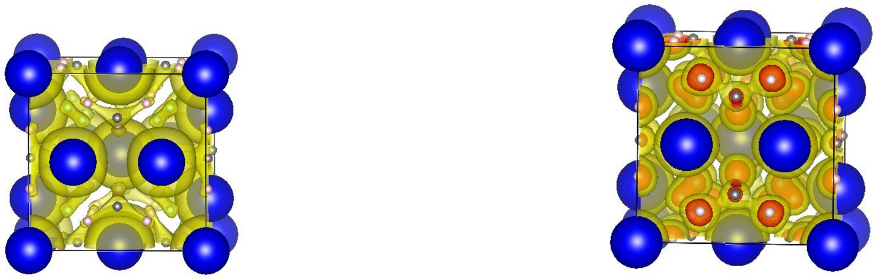

Figure S14: a) ELF isosurface (ELF=0.32) for the pure $Pm\bar{3}n$ Ba sublattice of $BaH_{5.75}$ at 50 GPa (in yellow). Ba atoms represented as blue spheres and ELF maxima as green, pink and grey spheres. b) ELF isosurfaces, ELF=0.95 (in orange) and ELF=0.65 (in yellow) for $Pm\bar{3}n$ $BaH_{5.75}$ at 50 GPa. Ba atoms represented as blue spheres and the 3 non-equivalent H atoms as green, pink and grey spheres, respectively.

S5 Other BaH_x compounds

Cmcm-H3 is an interesting structure where we identify H_3 units (H (8*f*)-H $(4c)_1$-H (8*f*)). In this structure, Ba donates electrons to both, $H(4c)_2$ and the H_3 units, that hold similarly high negative charges. This structure should be understood as $BaH^-(H_3^-)$.

We have also calculated the ELF value at the middle H-H point for H-H distances up to 1.5 Å for the high-symmetry structures $BaH_{5.75}$ and the high-pressure superconductor BaH_{12} (Sup. Fig. S15). There is a clear gap that allows to identify molecular units.

Table S2: Bader charges and ELF basin populations for the *Cmcm*-H3 structure at 50 GPa (a=3.462 Å. b=7.740 Å, c= 5.080 Å).

Atom	x	y	z	Bader charge	ELF charge
Ba (4*c*)	0.000	0.117	0.250	+1.164	+1.485
H $(4c)_1$	0.000	0.402	0.250	+0.085	+0.320
H (8*f*)	0.000	0.610	0.565	-0.354	-0.540
H $(4c)_2$	0.000	0.810	0.250	-0.541	-0.732
H_3				-0.623	-0.754

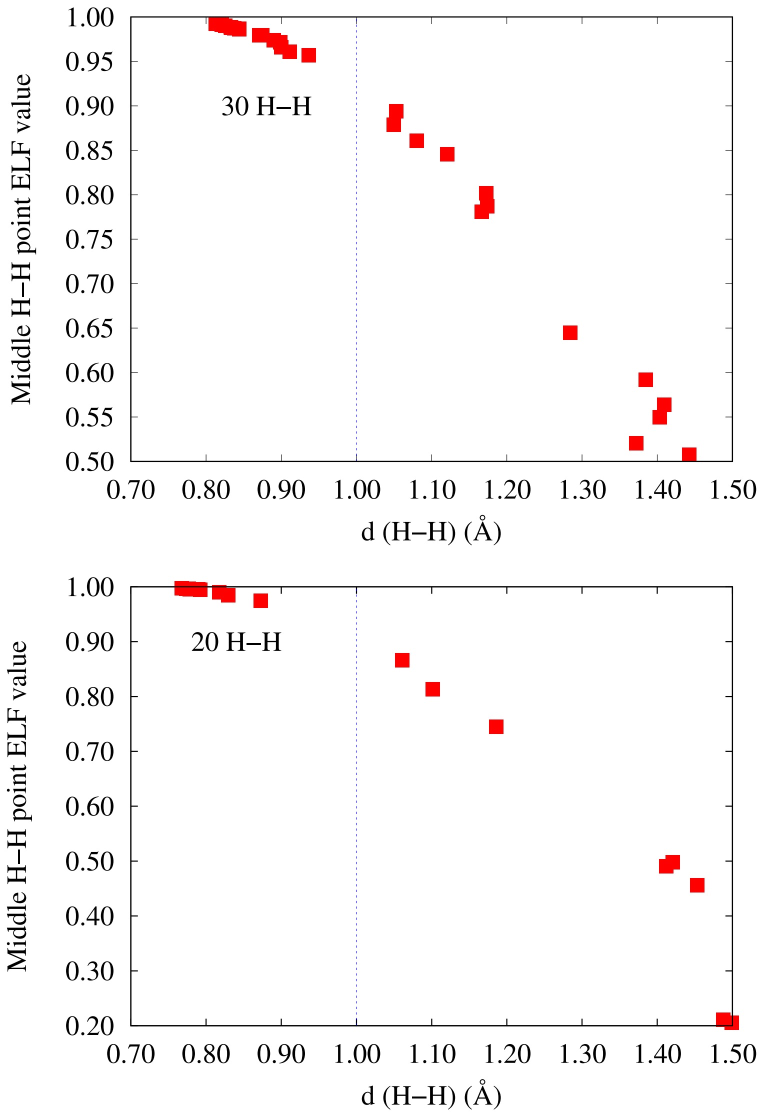

Figure S15: ELF value at the middle H-H point for H-H distances up to 1.5 Å at 50 GPa. a) $BaH_{5.75}$, b) BaH_{12}

S6 Details of Molecular Dynamics of specific materials

S6.1 MD of $I4/mmm$ BaH_4

We ran a series of MD simulations on BaH_4 with high and low c/a ratios, at 50GPa and 200GPa, and at 300K, 600K and 1000K.

At 50GPa/300K, (Sup. Fig.S8) the I4/mmm simulation box transformed to a distorted structure, which after relaxation has a cell (a = 7.078 , α = 86.9, b = 7.593 , β = 89.6, c = 10.624 , γ = 101.7)

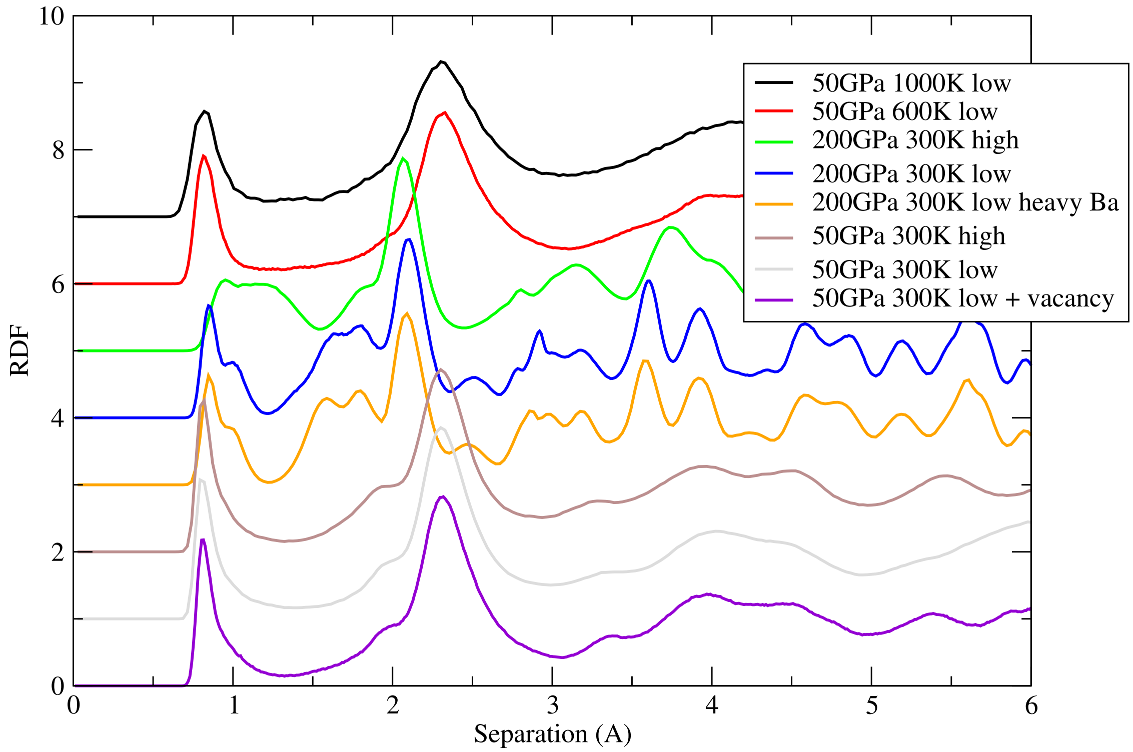

Figure S16: Radial distribution functions from molecular dynamics simulations in $I4/mmm$ BaH_4 at various conditions. Each graph shows total g(r) normalised to total atomic density.

this contrasts with molecular dynamics with ”correct” Ba mass where the Ba motion was too slow to allow for the transformation and rotation of the hydrogen molecules was observed. A distinctive feature of this structure is the H_3^- units, as shown in figure S8. This type of “polyhydrogen” chain has been associated with insufficient k-point sampling,[15] but here it is stable in grids up to 12x12x6 in an 80-atom supercell. In the RDF (Sup. Fig.S16) these units manifest as a shoulder on the first H_2 peak. In figure S8 we show that these H_3^- units in the relaxed structure are largely parallel, but including two which have different orientations. The H_3^- units were previously noted in a metastable $C2c$ structure, so the relaxed MD structure appears to be this structure with misoriented defects. The bond-breaking analysis detects many bond breaking events, but on closer inspection these are almost all within the H_3 triplets. This means of bond-count is remarkably stable since both H_3^- units and H_2 molecules contain 2 atoms with one neighbour, so if a third atom approaches a molecule, or an H_3^- unit breaks, the measure is unchanged.

For same 50GPa/300K conditions starting with high c/a=2.323, the c/a ratio remained roughly

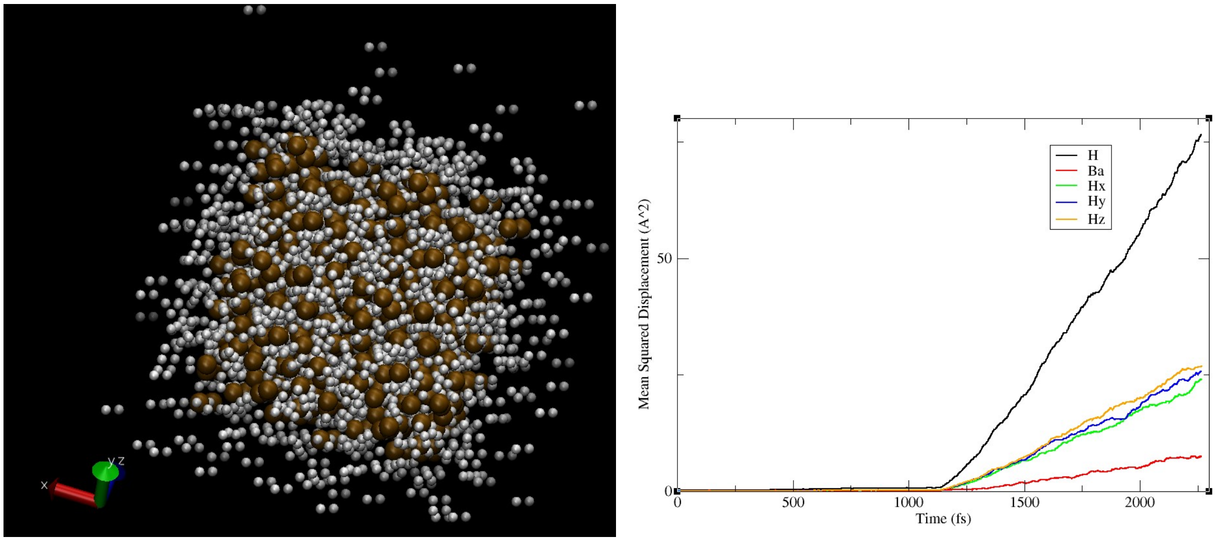

Figure S17: **Equal-mass MD can be used to demonstrate melting : molten Ba** Details from the "equal-mass" MD simulation in $BaH_{5.75}$ which melts at 1000K 100GPa a) Snapshot of the molten configuration (doubled cell, periodic boundary conditions suppressed). b) Mean squared displacements of the "light" Ba (red) and Hydrogen (black, also x,y,z components showing homogeneous motion)

constant. Despite this, the radial distribution function is remarkably similar to the low c/a version, dominated by H_2 molecules, with an H_3^- shoulder below 1 Å and the Ba-H peak at around 2.3 Å.

At 200GPa/300K the molecules, initiated along the z-direction, rotated into the x-direction and rebonded, the simulation box remained close to orthorhombic. The H_2 molecules retained their integrity, although in the relaxed final configuration each H_2 bond (0.87 Å, Mulliken bond population $\equiv$ 1) is associated with a third hydrogen (1.02 Å, Mulliken bond population $\equiv$ 0.5). These objects seem to be intermediate between the symmetric H_3 units and independent H_2 and H^-. At these pressures the molecular rotation is strongly suppressed, giving rise to a much more structured radial distribution function. The high c/a version RDF is completely different the H_2 peak is poorly defined and now at 0.95 Å. Bond lifetimes are half that of the low c/a version

At 50GPa/600K the molecules started to rotate and c/a ratio dropped abruptly from the static value of 1.76 to 1.32, with $b < a$ by about 10%. Taking the mean of 2c/(a+b) across the run gives 1.41, the ratio at which the Ba atoms lie on an fcc lattice.

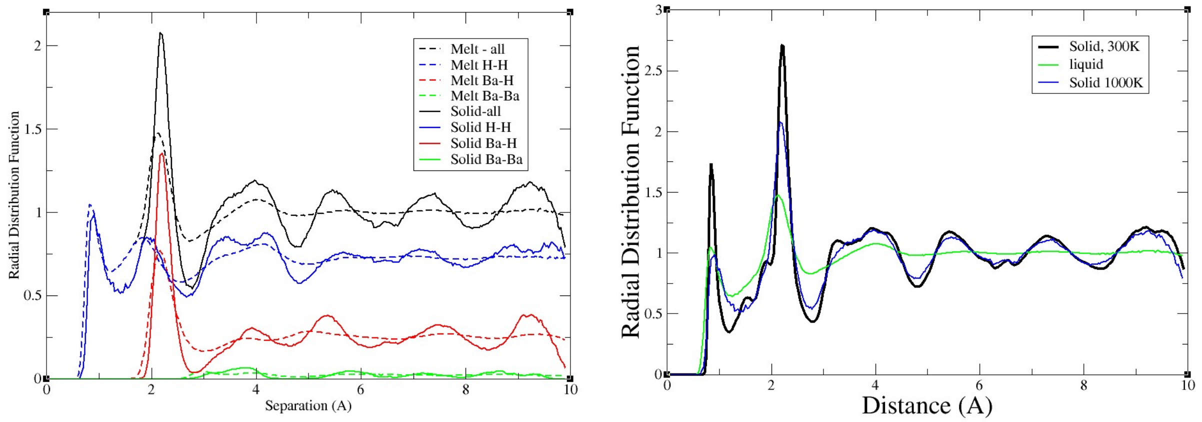

Figure S18: **$BaH_{5.75}$ as $BaH_2(H_2)_x$** (left) Radial distribution function of the 100GPa/300K simulation, compared with those at 1000K in liquid and solid form. (right) Pre- and post-melting radial distribution functions, and the breakdown by species. Analysis of the bonding shows that the MD in typically 94% fully bonded, compared to the low symmetry, relaxed structure $BaH_2(H_2)_x$ (227 compared to the ideal 240/368 hydrogens in bonds). The lifetime of these bonds, as measured by counting changes in the bond list, is given in Table 3.

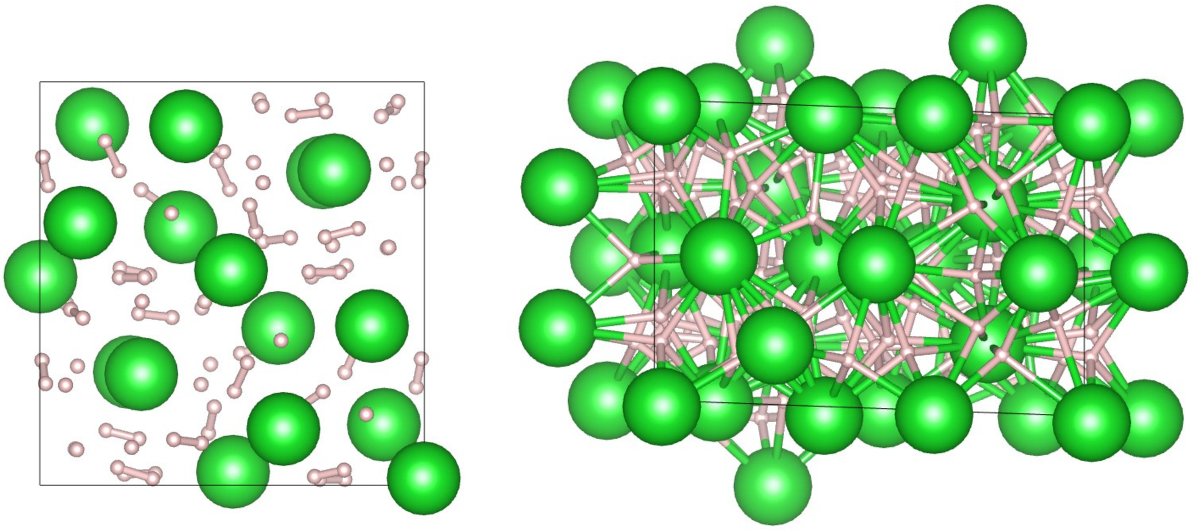

Figure S19: The most stable found structure (Pc - $Ba_{16}H_{92}$) of $BaH_{5.75}$ relaxed at 50GPa (left) Showing 30 H_2 bonds pfu in range 0.813-0.936Å. (i.e. $BaH_2(H_2)_x$ - next shortest is 1.049Å. (right) Showing Ba-H bonds less than 3Å, illustrating that each hydrogen has precisely 4 Ba neighbours (i.e. is confined to a tetrahedron).

S6.1 MD of other BaH_4 candidates

MD simulation in Cmcm-H2 at 50GPa shows this structure remaining stable for at least 5ps, with well-defined H_2 and H^- (Sup. Fig. S20) . Nevertheless, there are hints of the asymmetric H_3^-,

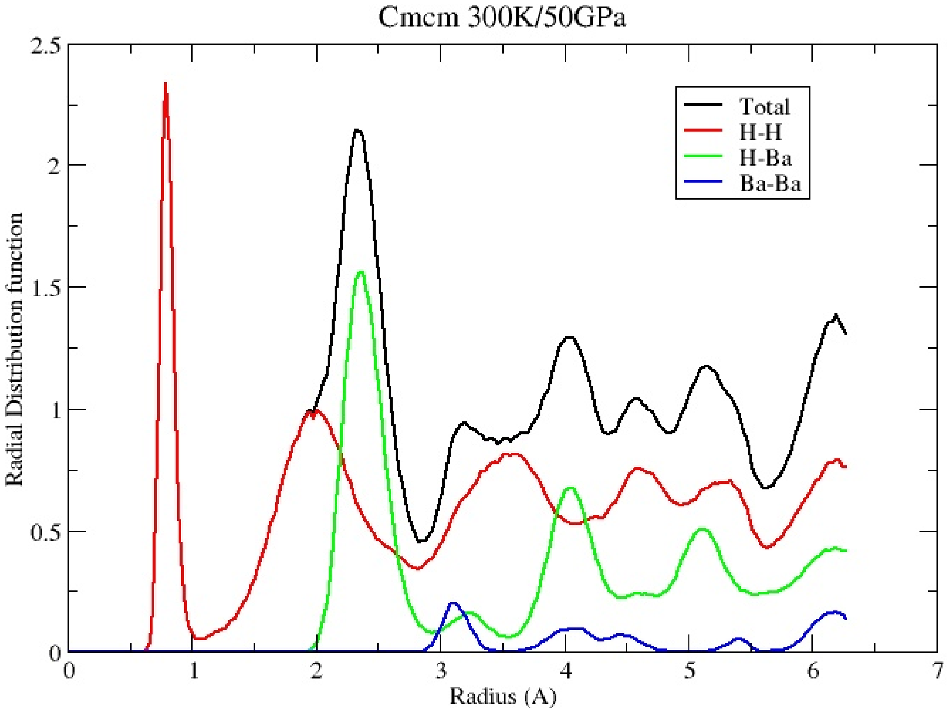

Figure S20: Radial distribution functions from molecular dynamics simulations starting in the *Cmcm*H2 structure. Each graph shows total g(r) normalised to atomic density, and contributions from each bond type.

with about 10% of H_2 dimers being associated with a third H within the first minimum of the radial distribution function. Likewise, the Cmcm-H3, started in the stable state with H_3^- units remains very close to the original structure, with 32 molecule throughout. Our bond-breaking analysis marks this structure as having frequent bond breaking, but this is largely an artefact of fructuations in the H_3^- units, which have mean bondlengths above 0.9Å and are therefore susceptible to H-H distances greater than 1Å appearing in thermal fluctuations.

S6.2 MD of $BaH_{5.75}$

The experimentally-observed $BaH_{5.75}$ $Pm\bar{3}n$ structure is stabilized by temperature, static relaxation calculations suggest a symmetry breaking distortion is which the ”atoms” pair up to become

molecular. At 50GPa, the minimum energy structure has only Pc symmetry, with a clear separation between the 15 H-H separations below 0.94Å and with bond population above $0.73e^-$, and the 16th closest dihydrogen pair (1.05Å/$0.5e^-$). These numbers demonstrate that in conventional chemistry terms the Ba_8H_{46} structure could be regarded as a solid solution $8BaH_2$+$15H_2$, rather than a Barium hydride. Further evidence for this comes from the electronic density of states which strongly resembles that in molecular I4mmm BaH_4, with distinctive bands associated with low-lying Ba 5s (-22eV) and 5p orbitals (-10eV), the H_2 molecular bonding orbitals. Closest to the Fermi energy are the hybridised bands of the Ba 5d and atomic hydrogen: population analysis in atomic basis set suggests that the Ba 6s are unoccupied.

The Weaire-Phelan topologically close-packed $BaH_{5.75}$ phase was run in an MD simulation with 64 formula units ($Ba_{64}H_{368}$ at 100GPa at 300K with full-mass Ba and 100GPa at 700K and 1000K with equal-mass MD. The 700K run was continued at 0 GPa. Molecular dynamics of the $Ba_{64}H_{368}$ supercell (100GPa/300K) reveals a different picture from the static case (Sup. Figs.S17 and S18). The molecularization to approximately $Ba_{64}H_{128}(H_2)_{120}$ occurs, but the covalent bonds are continuously breaking and reforming. The topological close packing means that all interstitial regions are tetrahedral, and there is precisely one hydrogen atom in each tetrahedron; However, the hydrogens are able to pair up with covalent bonds forming through the triangular faces of the tetrahedra, and analysis shows that on average 114 bonds exist at any time. This number follows a skewed distribution with a maximum at 120: as expected for x from the formula $(BaH_2)(H_2)_x$. The radial distribution function does not drop to zero, and so this is somewhat sensitive to R_{cut}. With 368 hydrogen atoms in the cell, on average there are 133.5 unbonded H units compared to 128 expected in the relaxed ground state structure. The simulation ran for 6.5ps during which time all hydrogen atoms in the cell spent some time as molecules, most forming bonds at some point with more than one of the four atoms in neighbouring tetrahedra. No hydrogen atom ”escaped” from its original tetrahedron.

Heating to 700K with the equal mass sampling gives a similar result, while continuing the simulation at 0GPa/700K reduces the rebonding and increases the molecule count to an average of

119.

A longer run with 100GPa/1000K melted after a lengthy equilibration period. The melt structure has a rather similar first peak to the solid H-H radial distribution, showing that the majority of hydrogen is still in molecular form: the exact number depends of the radius chosen for a molecules. A distinct Ba-H rdf peak is evident around 2.2Å in both liquid and solid, but it is less distinct in the liquid. The Ba-Ba liquid rdf has an unusual double peak at 2.8Å and 3.9Å This is quite unlike the solid. We can associate the first peak with adjacent Ba^{2+} ions (textbook ionic radius 1.35Å), and the second peak with two Ba's separated by hydrogen. It is notable that, despite the low Ba mass, the mean-squared displacement of the Ba is still much lower than that of hydrogen, indicating the role of size in diffusion.

We attempted to determine stable ground-state structure by relaxing MD snapshots. Relaxed structures were always significantly more stable than the $Pm\bar{3}n$ structure reported from X-ray diffraction, although the Ba atoms are always close to that structure (Fig.S19).

Relaxing a snapshot from the large MD at 100GPa to 0GPa gave precisely 120 bonds by the sub 1Å criterion, with a gap in observed bondlengths between 0.92 and 1.26Å, coinciding with a sharp drop in the Mulliken bond charge from 0.8 to 0.2.

Relaxing to 50GPa had a similar effect, with slightly longer bonds and a less sharp division between bonded and unbonded. Again, the Mulliken charge drops from 0.8 to 0.2 between 0.9Å and 1.2Å, but now there are several bonds with distances (and charges) between.

Other snapshots showed similar results, but no unique structure emerged. It is evident from the relaxed structures that the H_2 molecules are oriented perpendicular to the vector the short BaH vector. This follows from the fact that the bond links hydrogens in different tetrahedra, and therefore goes through a triangle of three bariums.

These unit cells are too large for lattice dynamics calculation of Raman spectra, but we can get some indication of the vibrations in the system from the velocity autocorrelation function (Sup. Fig.S21. This shows a large bump between 300 and 1800 cm^{-1} corresponding to lattice and librational motion, and a broad shoulder extending up to 3300 cm^{-1}. This breadth is due to both the

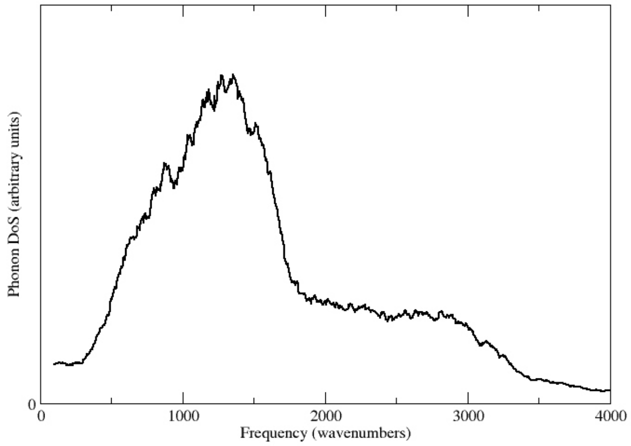

Figure S21: Fourier transform of the velocity autocorrelation function from MD of the $BaH_{5.75}$ structure (100GPa/300K). The H_2 vibrations show a broad but unresolvable region between 2000 and 3000 wavenumbers.

range of H_2 environments, giving rise to multiple vibrational frequencies, and their short lifetime which broadens each peak.

S6.3 MD of BaH_{12}

BaH_{12} was reported by Chen et al. to be a molecular metallic barium hydride, identified and pseudocubic, and that ab initio calculations show that newly discovered semimetallic BaH_{12} contains H_2 and H_3 molecular units and detached H chains which are formed as a result of a Peierls-type distortion of the cubic cage structure.

We ran molecular dynamics at 300K/250GPa 300K/150GPa and 300K/50GPa and found it to be stable (Sup. Figs. S22, S23 and S24 We used a supercell containing 8 formula units which in principle allows up to 48 molecules to form. For this high hydrogen concentration bond making and breaking occurred frequently, invariably via an H_3 transient. Once again, the material is revealed as $BaH_2(H_2)_x$. Typical calculations with Ba_8H_{96} generate precisely 40 molecules. The sharp H-H molecular peak is present at all pressures, becoming notably less sharp, and developing a pronounced tail at higher pressures. The second peak, corresponding to non-bonded H-H, is

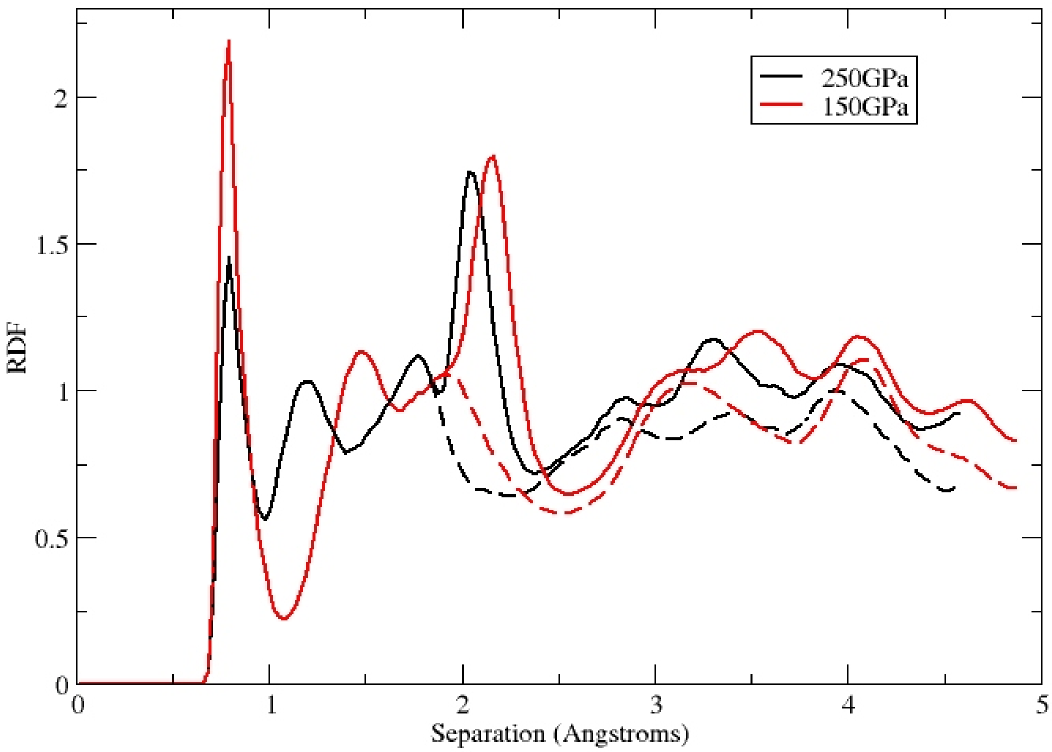

Figure S22: Radial distribution functions from molecular dynamics on BaH_{12}. Solid lines are total RDF, dashed lines count only the H-H distances. The sharp first peak at 0.8Å indicates the preferred molecular bondlength seen in these simulations and is almost independent of pressure. The compression with pressure is evident only in the second peak - the intermolecular distance. The second largest peak is the BaH nearest-neighbour separation, which can be seen to reduce with pressure. The overall crystal structure can be described as pseudocubic.

distinctive and moves to shorter distances with pressure, as does the BaH separation.

There is some slow but significant diffusion of hydrogen (MSD at $\equiv 2\AA$ in 5ps). Interestingly, the diffusion is slowest at 50GPa, and fastest at 150GPa, with 250GPa being intermediate suggesting that both the rate of bond breaking and the space available for rotation are important.

The mean squared displacement plateaus, as expected for a solid. The Ba atoms move very little from their lattice sites (around 0.1Å), despite the reduced mass. However theBarium hydrogens are fairly mobile, with a MSD around 1.5Å . This is consistent with making and breaking of bonds,

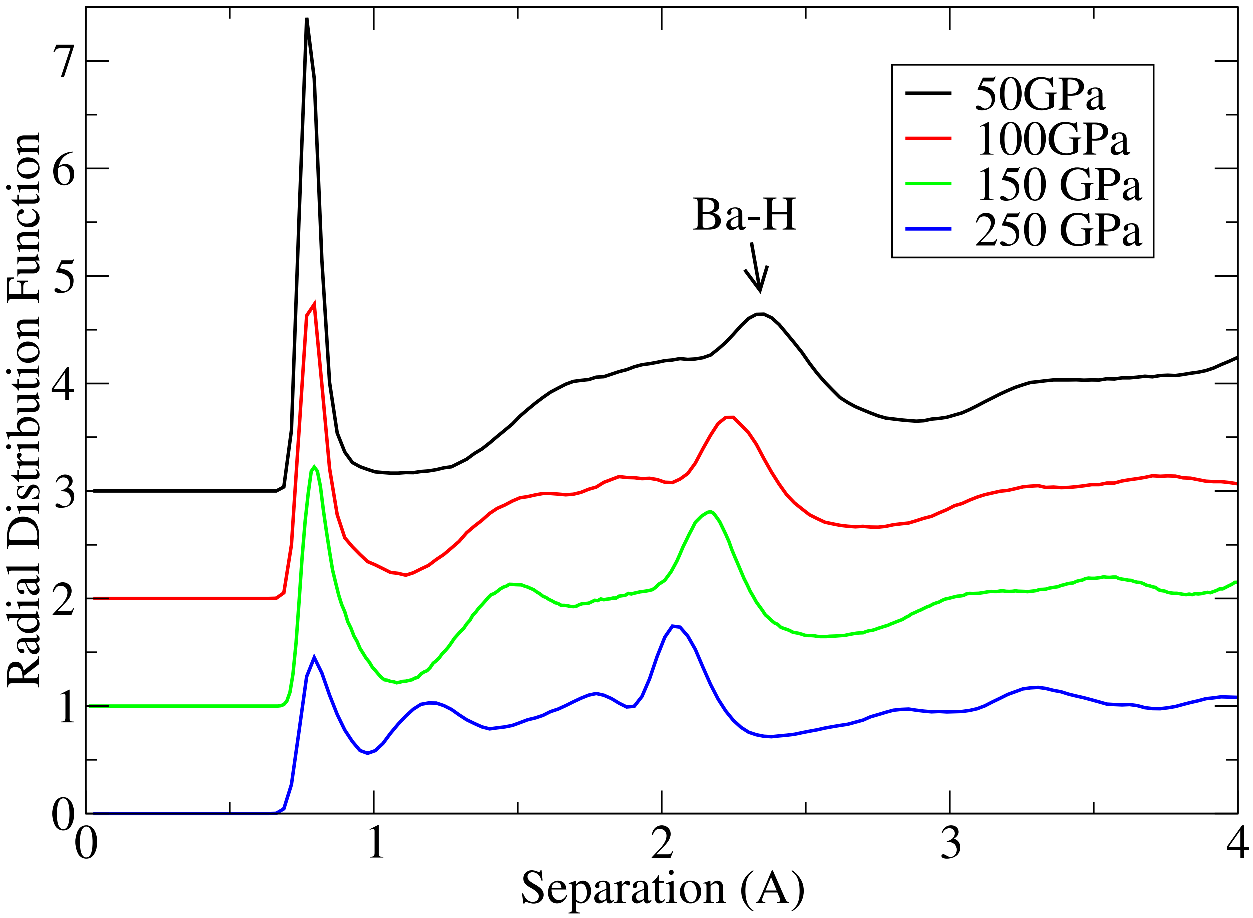

Figure S23: Radial distribution functions at all pressures considered (300K) from molecular dynamics on BaH_{12}.

plus molecular rotations. Detailed analysis of the trajectories using vmd and inspection of lists of neighbours shows a slow diffusion of hydrogens at a timescale of tens of picoseconds associated with $H+H_2 \rightarrow H_3 \rightarrow H_2+H$ processes followed by molecular rotation.

We used the *'Atoms with precisely one neighbour within R_{cut}=1Å '* criterion to define molecules. If molecules were forming randomly, one would expect to see a Gaussian distribution about some average value, with strong dependence on R_{cut}.

In 10000 timesteps of MD with Ba_8H_{96} at 150GPa/300K, 91% of snapshots showed 40 molecules, 8% showed 39 and 1% showed 38. Only at one timestep (0.01%) was a 41 molecule configuration reported. The sub-40 molecule configurations typically lasted for a few fs, indicating that they are a fast physical process rather than noise. The precise number of non-40 molecule snapshots is

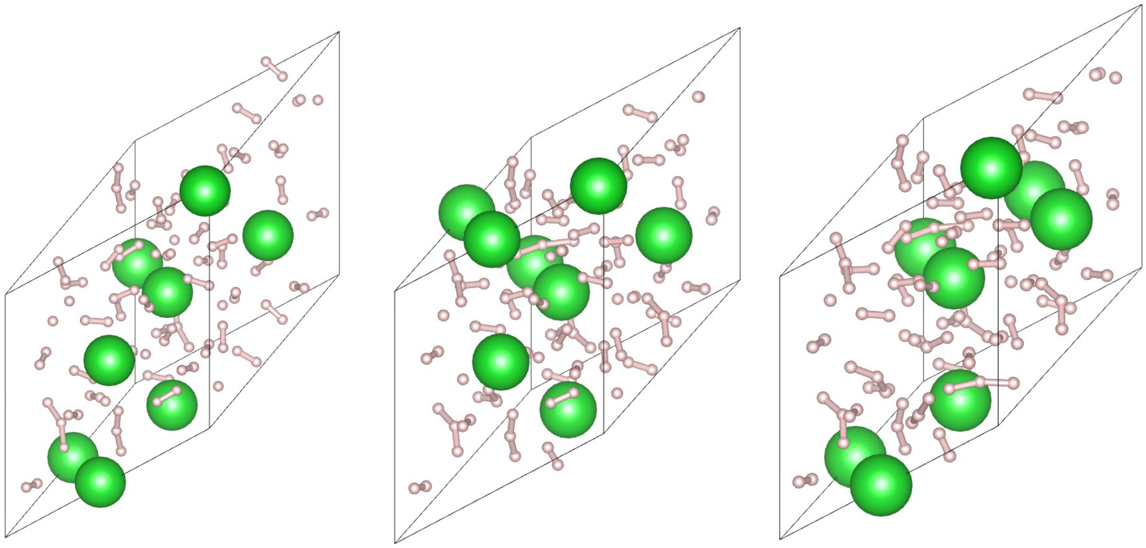

Figure S24: **BaH_{12} as $BaH_2(H_2)_5$** Relaxed P1 BaH_{12} structures at 50, 100 and 150GPa (left to right) Note the 100GPa case two Ba atoms have moved across the periodic boundary conditions. Analysis of molecular dynamics simulations from this structure reveals that the number of "H_2 bonds" (criterion: atoms with one neighbour within 1Å) is 5 per formula unit. This conclusion is remarkably robust to reasonable choice of the bondlength. By contrast, the more-easily calculated bond criterion "number of H-H distances less than 1Å " is very sensitive to the choice of cutoff. This is because there is a significant proton exchange via the $H_2+H^- \rightarrow H_3^- \rightarrow H^- + H_2$ mechanism.

sensitive to the choice of R_{cut}, but the massive preponderance is of $BaH_2(H_2)_5$ configurations.

At 250GPa, the bond-breaking is more frequent, with 40 molecules appearing only 16% of the time, but still 99% of snapshots show between between 36 and 40 neighbours.

In Chen's paper they present results for fcc, I4/mmm, and Cmc21 structures, although the most stable of their presented structure is pseudocubic $P2_1$. In molecular dynamics calculation we find many snapshots with lower enthalpy, all with P1 symmetry and $BaH_2(H_2)_5$ character. H_3^+ units are observed transiently at high T, and can be captured in relaxed structures. We find no evidence for longer chains or hydrogen molecular units with more than three atoms.

S7 Relation to superconductivity

There has been a tremendous interest in high temperature superconductivity in polyhydrides. This follows from Ashcroft's observation that metallic hydrogen could become a high-Tc superconductor.[16] The pressures required to make metallic hydrogen mean this has yet to be demonstrated. However, many hydrogen-rich compounds have shown high Tc superconductivity at achievable pressures. When hydrogen is mixed with a cation which donates electrons either to an atomic hydrogen conduction band (favourable) or a molecular antibonding orbital (unfavourable). In this way the molecular-atomic transition moves to lower pressures. However, as in BaH_4, the atomic form is much denser than the molecular form, so pressure is an essential stabilising factor via the PV term in the Gibbs free energy.

The rationale for high Tc polyhydride superconductors is that the temperature required to break the electron-phonon coupling is related to the phonon energy, and light hydrogen atoms leads to high phonon frequency and energy.

This leads to a dichotomy at low pressures. The hydrogens need to be close enough to form an extended phonon, but not so close that they form H_2 molecules. Another is that the atomic hydrogens in the conduction band are spaced by the hydrogens in molecules. These molecules nevertheless play a significant role in the Cooper-pair forming lattice phonons. BaH_4 illustrates this mechanism: here the molecules are H_2 but other spacer molecules are possible provided their vibration couples to the lattice phonon.

A final consideration is that the molecule formation is an alternative electron-pairing mechanism to Cooper-pair formation. In both cases the associated band gap opening stabilises the structure. In our Born-Oppenheimer MD there is no quantum coupling between electronic and nuclear degrees of freedom, so if the superconducting state was lower in energy than the molecularised one, we would not know.

References

(1) Bader, R. F. W. Atoms in Molecules: A Quantum Thory. **1994**,

(2) Becke, A. D.; Edgecombe, K. E. A simple measure of electron localization in atomic and molecular systems. *J. Chem. Phys.* **1990**, *92*, 5397–5403.

(3) Savin, A.; Nesper, R.; Wengert, S.; Fässler, T. F. ELF: The electron localization function. *Angewandte Chemie International Edition in English* **1997**, *36*, 1808–1832.

(4) Clark, S. J.; Segall, M. D.; Pickard, C. J.; Hasnip, P. J.; Probert, M. I.; Refson, K.; Payne, M. C. First principles methods using CASTEP. *Zeitschrift für kristallographie-crystalline materials* **2005**, *220*, 567–570.

(5) Perdew, J. P.; Burke, K.; Ernzerhof, M. . *Phys. Rev. Lett.* **1996**, *77*, 3865.

(6) Perdew, J. P.; Zunger, A. Self-interaction correction to density-functional approximations for many-electron systems. *Physical Review B* **1981**, *23*, 5048.

(7) Perdew, J. P.; Zunger, A. Self-interaction correction to density-functional approximations for many-electron systems. *Physical Review B* **1981**, *23*, 5048.

(8) Becke, A. D. Density-functional exchange-energy approximation with correct asymptotic behavior. *Physical review A* **1988**, *38*, 3098.

(9) Lee, C.; Yang, W.; Parr, R. G. Development of the Colle-Salvetti correlation-energy formula into a functional of the electron density. *Physical review B* **1988**, *37*, 785.

(10) Otero-de-la Roza, A.; Johnson, E.; Luana, V. Critic2: A program for real-space analysis of quantum chemical interactions in solids. *Comput. Phys. Commun.* **2014**, *185*, 1007.

(11) Mostofi, A. A.; Yates, J. R.; Pizzi, G.; Lee, Y.-S.; Souza, I.; Vanderbilt, D.; Marzari, N. An updated version of wannier90: A tool for obtaining maximally-localised Wannier functions. *Computer Physics Communications* **2014**, *185*, 2309–2310.

(12) Bonev, S.; Ashcroft, N. Hydrogen in jellium: First-principles pair interactions. *Physical Review B* **2001**, *64*, 224112.

(13) Ahuja, R.; Eriksson, O.; Wills, J. M.; Johansson, B. Theoretical high-pressure studies of caesium hydride. *Journal of Physics: Condensed Matter* **1998**, *10*, L153.

(14) Bi, T.; Zurek, E. Electronic structure and superconductivity of compressed metal tetrahydrides. *Chemistry–A European Journal* **2021**, *27*, 14858–14870.

(15) Magdău, I. B.; Ackland, G. J. Charge density wave in hydrogen at high pressure. Journal of Physics: Conference Series. 2017; p 042058.

(16) Ashcroft, N. W. Metallic hydrogen: A high-temperature superconductor? *Phys. Rev. Lett.* **1968**, *21*, 1748.

Table S3: Details of molecular dynamics simulations. WP is the Weaire-Phelan structure, Mass refers to the cation, bonds is the average number of hydrogen atoms within 1Å of precisely (one,two) other atoms. Broke is the number of times and atoms move into or out of the 1Å range of another, and this is normalise with time and number of bonds to give a number of such "events" per bond per ps. Relating this to the lifetime of the bond is complicated. e.g. harmonic bond oscillating beyond 1Å will record four "events" (two atoms, making and breaking) without being a genuine bond breaking. The common proton exchange process also records four "events" (one atom makes a bond, one breaks a bond, the central atom both makes and breaks). However in most of the recorded H_3 creation events, the "reaction" is "unsuccessful" and the initially-atomic hydrogen leaves as an atomic hydrogen. Taken together, a rough estimate is that bond breaking occurs with a frequency one order of magnitude less than the "Event" rate. Bondlength is the position of the first peak in the radial distribution function.

System	Time (ps)	T (K)	P (GPa)	Mass	bonds	Events N,ps^{-1},	Bond
Cmcm-H2 ($Ba_{16}H_{64}$)	2.5	600	50	4	32,0	715 $\Longrightarrow$ 4.5	0.80
Cmcm-H2 ($Ba_{16}H_{64}$)	10	300	50	4	32,0	12 $\Longrightarrow$ 0.01	0.79
Cmcm-H3 ($Ba_{16}H_{64}$)	5	300	50	4	32,7	16317 $\Longrightarrow$ 25.5	0.91
$I4/mmm$ (high, $Ba_{16}H_{64}$)	5.6	300	200	2	27,2	21058 $\Longrightarrow$ 69.2	0.95
$I4/mmm$ (high, $Ba_{16}H_{64}$)	5.0	300	50	2	32,2	6080 $\Longrightarrow$ 19.0	0.81
$I4/mmm$ (low, $Ba_{16}H_{64}$)	5.0	300	200	2	32,6	12154 $\Longrightarrow$ 38.0	0.84
$I4/mmm$ (low, $Ba_{16}H_{64}$)	4.4	1000	50	2	32,1	8901 $\Longrightarrow$ 15.8	0.80
$I4/mmm$ (low, $Ba_{16}H_{64}$)	3.9	600	50	2	32,6	4590 $\Longrightarrow$ 18.4	0.82
$I4/mmm$ (low, $Ba_{16}H_{64}$)	7.0	300	50	2	32,0	8019 $\Longrightarrow$ 15.9	0.81
$I4/mmm$ (low, $Ba_{16}H_{63}$)	4.8	300	50	2	31,1	5805 $\Longrightarrow$ 19.5	0.81
WP($Ba_{64}H_{368}$)	6.5	300	100	137	227,10	75761 $\Longrightarrow$ 25.7	0.85
WP($Ba_{64}H_{368}$)	0.5	700	100	2	227,10	4462 $\Longrightarrow$ 19.7	0.80
WP($Ba_{64}H_{368}$)	0.3	700	0	2	238, 0	1538 $\Longrightarrow$ 10.8	0.76
WP($Ba_{64}H_{368}$)	1.2	1000	100	2	175,10	36685 $\Longrightarrow$ 67.4	0.89
liquid ($Ba_{64}H_{368}$)	4.5	1000	100	2	202,17	80194 $\Longrightarrow$ 44.1	0.83
WP($Ba_{16}H_{92}$)	5.0	300	50	2	59,1	7514 $\Longrightarrow$ 12.8	0.82
Ba_8H_{96}	2.1	300	50	2	80,1	1855 $\Longrightarrow$ 5.5	0.77
Ba_8H_{96}	2.1	300	100	2	80,4	3526 $\Longrightarrow$ 10.6	0.78
Ba_8H_{96}	5.0	300	150	2	80,8	10387 $\Longrightarrow$ 13.0	0.78
Ba_8H_{96}	7.0	300	250	2	77,8	26254 $\Longrightarrow$ 24.4	0.80
